# Infection risks of city canal swimming events in the Netherlands in 2016

**DOI:** 10.1371/journal.pone.0200616

**Published:** 2018-07-27

**Authors:** A. D. Hintaran, S. J. Kliffen, W. Lodder, R. Pijnacker, D. Brandwagt, A. K. van der Bij, E. Siedenburg, G. J. B. Sonder, E. B. Fanoy, R. E. Joosten

**Affiliations:** 1 Public Health Service Utrecht region, Zeist, the Netherlands; 2 Public Health Service Amsterdam, Amsterdam, the Netherlands; 3 Centre for Infectious Disease Control, Preparedness and Response Unit, National Institute for Public Health and the Environment (RIVM), Bilthoven, the Netherlands; 4 Centre for Infectious Disease Control, Epidemiology and Surveillance, National Institute for Public Health and the Environment (RIVM), Bilthoven, the Netherlands; 5 Department of Medical Microbiology and Immunology, Diakonessenhuis, Utrecht, the Netherlands; 6 Division of Infectious Diseases, Department of Internal Medicine, Academic Medical Center (AMC), University of Amsterdam, Amsterdam, the Netherlands; Universita degli Studi di Parma, ITALY

## Abstract

**Introduction:**

Swimming events in city canals are gaining popularity in the Netherlands, even though canal water is usually not officially designated for recreational use. Knowledge regarding the risk of infection after swimming in canals is limited. An outbreak was reported in 2015 following a canal swimming event in Utrecht, the Netherlands. Local governments were concerned about the health risks of such events. In order to assess the safety of canal swimming, the Public Health Service (PHS) prospectively investigated two city canal swimming events in 2015. In 2016, we repeated this study, aiming to prospectively determine the risks of infection during two urban swimming events, the Utrecht SingelSwim 2016 (USS) and the Amsterdam City Swim 2016 (ACS).

**Methods:**

We sent online questionnaires to 271 USS participants and 2697 ACS participants, concerning personal characteristics, symptoms, and exposure. Participants were asked to forward the questionnaire to three relatives, i.e., non-exposed. We analyzed water samples from the USS venue taken during the event, as well as stool samples of USS participants with acute gastrointestinal illness (AGI). AGI was defined as diarrhea and/or vomiting within seven days after the event. We calculated adjusted risk ratios (RR) for AGI in the exposed group compared with non-exposed respondents, using binomial regression models.

**Results:**

The questionnaire was returned by 160 USS participants (exposed) (59%) and 40 non-exposed relatives. Five percent of the exposed (n = 17) and 3% of non-exposed (n = 1) reported AGI (RR = 1.69; 95% CI: 0.23–12.46). Norovirus genogroup II was detected in two of six USS water samples and in none of the three stool samples. In one of three stool samples, rotavirus was detected.

The questionnaire was returned by 1169 ACS participants (exposed) (43%) and 410 non-exposed relatives. Six percent of the exposed (n = 71) and 1% of non-exposed (n = 5) reported AGI (RR 4.86; 95% CI: 1.98–11.97).

**Conclusion:**

Results of the ACS event showed a higher risk for AGI among the exposed, indicating that participants of events in urban canals in the Netherlands could be at a higher risk for AGI than those not participating. The inconclusive results from the USS are likely due to the small sample size. Swimming in non-monitored open water can bring health risks and more knowledge about environmental and human risk factors helps reduce the risk by being able to more specifically advise organizations and governments.

## Introduction

The Netherlands is a country known for the water in and around its cities. The typical Dutch city canals in the historical inner cities are also used as swimming event venues, in many cases charitable events promoting exercise and sports supported by local government.

City canals are not regulated and monitored following European Union bathing water regulations, as they are not official bathing sites [1]. City canals are usually a mixture of water from different supply sources: rivers, waters from rural areas, but also overflow of sewage water after heavy rainfall. In many Dutch cities, the canals are also used as recreational sites for tourist and private boats. Sources of contamination in the canals are mainly street dirt, sewage overflow, bird droppings, rats and mice, and in some cases discharge from houseboats. Therefore the canals can contain many pathogens of human and animal fecal origin [2]. For bathing water, the parameters to assess water quality are coliform bacteria (e.g., *Escherichia coli*) and enterococci levels in water [1]. The levels are related to fecal pollution, which is assessed by this method. One of the main aims of regulation is the prevention of gastrointestinal infections. The most commonly identified health problems after swimming in open water in the Netherlands are skin complaints, followed by gastrointestinal illness [3].

In advance of a swimming event in unofficial swimming water like a city canal, local authorities sometimes measure enterococci and *E*.*coli* levels to have a general estimate of the water quality. However, open water can contain many other pathogens, which are not measured, such as enteric viruses. These pathogens can be naturally present in aquatic environments, or introduced by human or animal fecal pollution [2,4]. It is known that coliform bacteria and enterococci levels are not always predictive for the presence and levels of other pathogens [5,6]. In addition, activities in open water can lead to (outbreaks of) gastrointestinal illness. Outbreaks have been reported at swimming events in recreational water as well as in other open non-official swimming waters [6–10].

In 2015, a triathlon event in the inner city waters of Utrecht, the Netherlands, led to an outbreak of acute gastroenteritis with 73 cases (attack rate 31%) [11]. Following this event, local governments asked public health services (PHS) about the safety and infection risks for future swimming events in canal waters. In order to assess their safety, the PHS investigated two city canal swimming events in 2015. One of the events, the Amsterdam City Swim in 2015, led to an outbreak of gastroenteritis with 427 cases (attack rate 31%), the second event reported 7 cases among swimmers (attack rate 9%) [12].

We repeated the previous study conducted in 2015 in Utrecht and Amsterdam. The results of the study conducted in 2015 were influenced by weather conditions where heavy rainfall led to sewage overflow, and are perhaps not representative for all urban canal swimming events held in Dutch summer weather conditions. Repeating the study using the same study protocol under possibly different weather conditions could underline known risk factors or reveal new risk factors for health complaints after such events.

The aim of this study was to determine risk factors of illness due to swimming in urban canal water to confer more targeted preventive measures and recommendations for organizers, municipal authorities, and participants of swimming events.

## Methods

### Study design

The study design is the same as that of the study conducted in 2015 [12]. Two prospective cohort studies were conducted among participants of two fundraising swimming events in city canals in the Netherlands: the Utrecht SingelSwim 2016 (USS) and the Amsterdam City Swim 2016 (ACS).

The USS was held in an inner city canal in Utrecht on 19 June 2016. A total of 271 participants swam distances of 800, 1,200, or 2,000 meters. People above 12 years of age who were not pregnant and did not have serious medical conditions could participate in the event.

The ACS took place in the city of Amsterdam on 11 September 2016. A total of 2,697 participants swam distances of 700 or 2,000 meters. People above 10 years of age who were not pregnant and did not have serious medical conditions could participate in the event. Children under the age of 15 years were only allowed to swim with adult supervision.

### Questionnaire

The event organizers informed all participants about the study before the event. A week after each event, the event organizers sent a request to all participants to fill in an online questionnaire (by NetQ (USS) and LimeSurvey (ACS)). Participants were asked to forward the questionnaire to three friends or relatives who had not taken part in the swimming event. The friends and relatives are considered the group of non-exposed people. The event organizers sent an e-mail with an openly accessible hyperlink to the questionnaire. The e-mail also stated that participation was voluntarily and that the questionnaire was accessible for 12 days after the e-mail was sent.

For analysis, we only included questionnaires that were completed.

In the questionnaire we asked respondents detailed information about their health complaints before and after the event, general health status, medical and travel history, dietary habits, known illness among family or friends, the use of catering, toilets and/or showers during the event, and specific details about their participation in the event (e.g., distance, duration, water ingestion, swimming technique, type of wetsuit, previous training in open water). Responses were categorized based on literature (e.g., mouthfuls of water, medication use) or to make them comparable with results from our previous study (i.e., age, number of sips, medication use) [12] (S1–S4 Files).

### Case definition

A case of acute gastrointestinal illness (AGI) was defined as a respondent with diarrhea and/or vomiting within seven days after the event. The case definition for skin complaints included rash, red spots, or signs of skin infection within the same period. Having a cold, coughing, or dyspnoea within seven days after the event was defined as a respiratory case. Cases were used to define prevalence of disease after the event.

### Laboratory and environmental investigations

**Fig 1:**
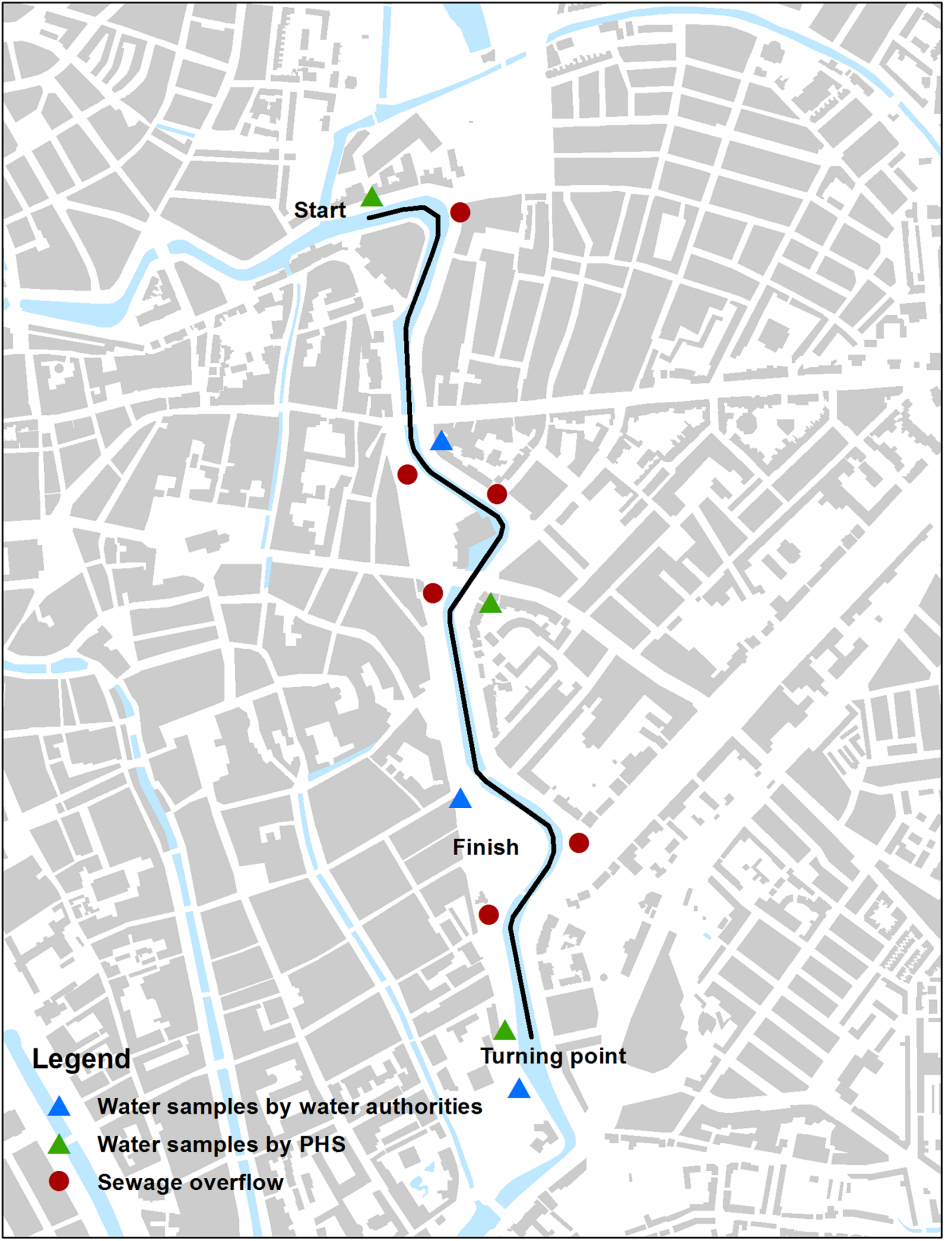
Swimming route, water sample locations, and sewage overflow locations of USS.

At the USS, two researchers collected water samples on the day of the event before and after the city swim. Samples were collected in empty and clean soda bottles of 1.5 L. Samples were taken at three different locations along the swimming route (start, halfway, and finish) at two different moments in time (before and after the race) (Fig 1). Following Dutch guidelines for public health diagnostics in infectious disease control, environmental samples would only be analyzed if two or more cases of illness were reported among participants. In our study, the samples were tested two weeks after the event. In the meantime the samples were stored at five degrees Celsius awaiting microbiological analysis.

The total of six water samples were filtrated through positively charged membrane filters and these filters were subsequently incubated with a Tris/Glycine/Beef extract buffer (pH 9.0). The resulting eluate was tested for the presence of norovirus genogroup I and II RNA with real-time Polymerase Chain Reaction (PCR) following the protocol and using the primers as described in another publication from the testing laboratory [13]. For PCR testing, approximately 9 ml of canal water was tested. Samples tested positive were further characterized by sequence analysis to potentially match with organisms found in stool specimen [14].

A total of six samples of 0.1L were tested with PCR for microbial agents *Campylobacter* species, Salmonella and by culture for *Yersinia species*, following diagnostic laboratory testing of feces [15]. For S*higella*, primers and probes targeting the ipaH gene were added.

At the USS event, local water authorities also collected water samples five days in advance, since they do not test waters every day. *E*.*coli* and enterococci levels were tested following EU guidelines (reference methods from the International Organization for Standardization (ISO) ISO 7899–1, ISO 7899–2, ISO 9308–3 and ISO 9308–1)[1,16–19]. Samples were taken at three different locations (Fig 1).

**Fig 2:**
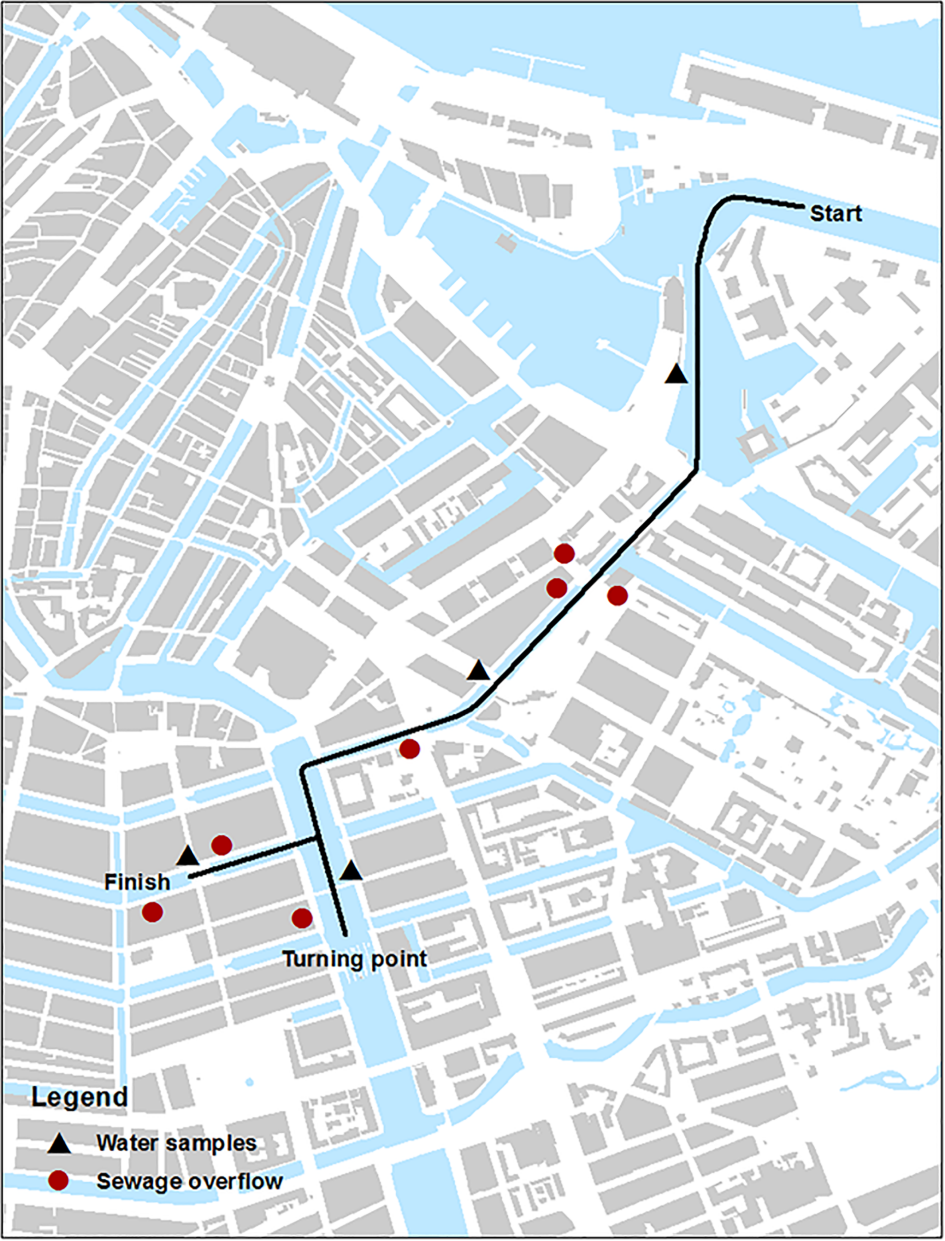
Swimming route, water sample locations, and sewage overflow locations of ACS.

At the ACS, only the local water authorities collected water samples. Samples were taken at four different locations along the route during the start of the event (Fig 2). The cut-off values used for interpretation of the results are 1800 cfu/100 mL for *E*. *coli* and 400 cfu/ 100 mL for enterococci in single sample measurements for both the USS and ACS, following Dutch water authorities guidelines for recreational water [20,21].

Persons who developed diarrhea within seven days after the USS event, were requested by phone to collect a stool sample if they provided their contact details. The number of days between onset of diarrhea and receipt of the stool sample at the laboratory was between 7 days and 14 days. A home-use stool sampling kit, which was provided by the Dutch Public Health Institute (RIVM), was sent by mail to persons with diarrhea, with a request to forward the kit to the diagnostic laboratory at RIVM within one day. The stool was tested by PCR for norovirus, sapovirus, rotavirus group A, adenovirus, and astrovirus. Stool specimen that tested positive for either norovirus or rotavirus were further characterized by sequence analysis [14] to potentially match with organisms found in water samples or with other stool specimen from other cases.

Exposed cases who developed diarrhea within seven days after the ACS were also requested to collect a stool specimen in an attempt to match these with pathogens found in water.

### Statistical analysis

Questionnaire data were analyzed using Stata Version 13 software [22]. Demographic differences between exposed and non-exposed were compared using students T-test and χ2-test for age, chronic disease, and gender. The prevalence of health complaints at both events between exposed and non-exposed was compared using a χ2-test or Fisher’s exact test if applicable. To compare AGI and risk factors in exposed with non-exposed, we calculated adjusted risk ratios (aRR) with corresponding 95% confidence intervals (95% CI) using log-binomial models. Thirty-seven variables were included (S5 File). Variables were categorized following the previous study to make them comparable with previous results (i.e., age, number of sips, medication use). Variables with p-value ≤ 0.2 in univariable analysis were included in a multivariable model, which was built in a backward stepwise manner. Variables gender and age were always retained in the model.

Additional univariable analysis and multivariable analyses were conducted among the exposed only to further investigate specific risk factors among participants of the swimming events. For the multivariable analyses we included variables with p-value ≤ 0.2 in the univariable analysis. It was built in a backward stepwise manner. Variables gender, age, and ingestion of water were retained in the model.

### Ethical review

The Medical Ethics Committee (MEC) of the University Medical Center Utrecht assessed the study and concluded its exemption from approval by them: reference number: WAG/mb/17/032094. Both the questionnaires and stool examination were approved by the MEC. No permission was needed for taking water samples since the water sites were open urban water. Participants and non-participants of the event were asked by e-mail to voluntarily participate by clicking the link in the e-mail.

The purpose of the study was explained in the introduction of the survey and respondents could decide at any point in the survey to discontinue.

## Results

### Utrecht SingelSwim cohort

A total of 160 USS participants filled in the questionnaire (response rate 59%). Relatives and friends who did not participate in the event returned 40 questionnaires.

**Fig 3:**
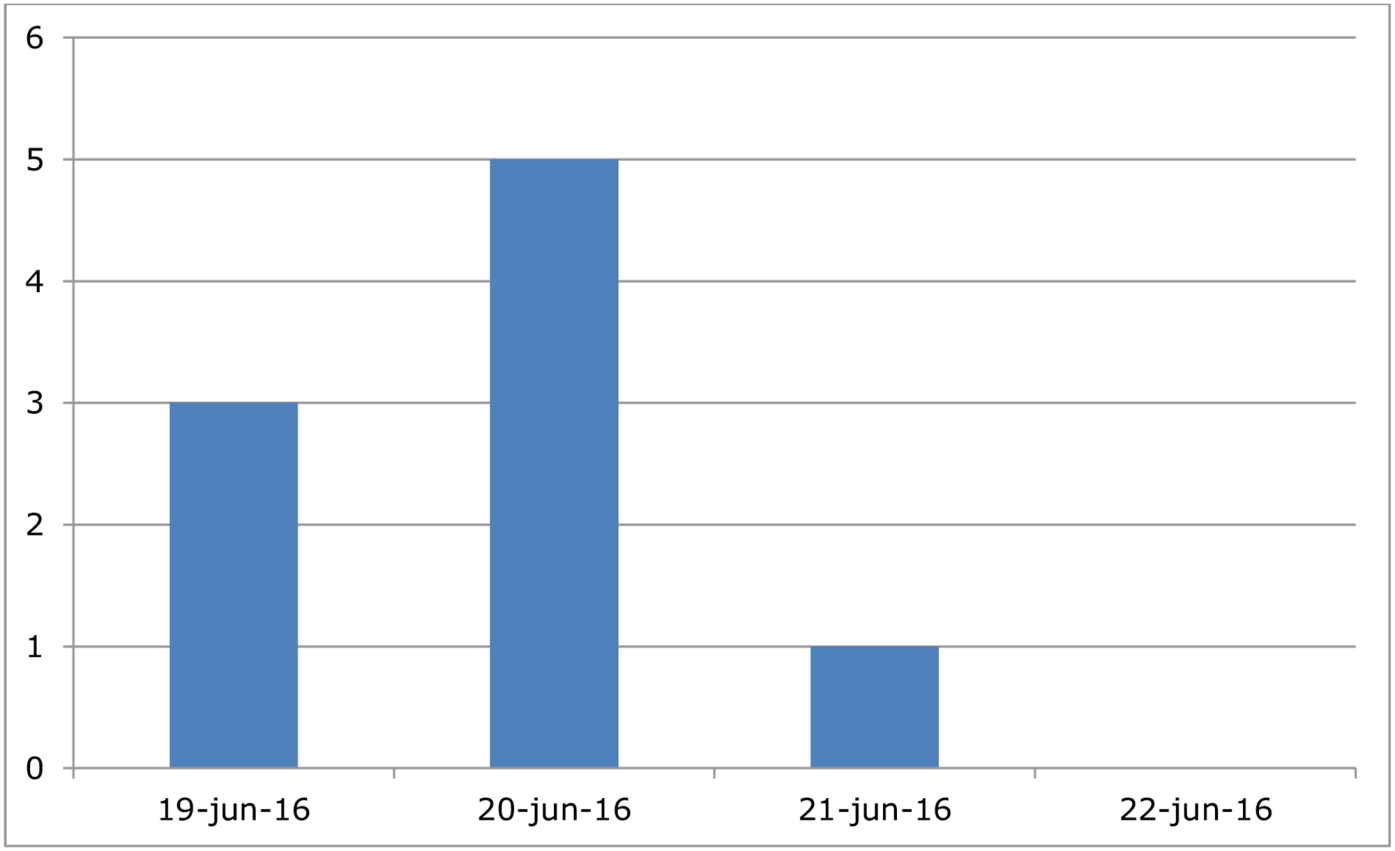
Epidemiological curve Utrecht SingelSwim (USS), 19 June 2016. The number of cases by date of onset of Acute Gastrointestinal Illness following the event.

In the USS, 40 (25%) of the exposed respondents reported any kind of health complaints (gastrointestinal, respiratory, or skin complaints), compared with 4 (10%) of the non-exposed respondents (p-value = 0.05). Comparison of demographic characteristics (age, presence of chronic disease, and gender) did not indicate a significant difference between exposed and non-exposed respondents (p-value = 0.62, p-value = 0.76 and p-value = 0.39, respectively). Cases of acute gastrointestinal illness, respiratory illness, and skin symptoms were compared between exposed and non-exposed (RR 1.7; 95% CI: 0.23–12.0 p-value = 0.61, RR 1.56; 95% CI: 0.36–6.70 p-value = 0.55, RR non calculable, respectively) (Table 1). Most of the AGI cases developed health complaints within one day after the event (Fig 3).

**Table 1:** Reported health complaints of questionnaire respondents of the Utrecht SingelSwim 2016.

	Exposed		Non-exposed		Univariable RR (95% CI)	P-value	Multivariable RR (95% CI)^a^	P-value
	(N = 160) Cases n	%	(N = 40) Cases n	%				
**Health complaints** ^b^								
Acute gastrointestinal illness	8	5.0	1	2.5	2.0 (0.26–16)	0.50	1.7 (0.23–12)	0.61
Respiratory illness	12	7.5	2	5.0	1.5 (0.35–6.4)	0.58	1.6 (0.36–6.7)	0.55
Skin symptoms	9	5.6	0	0	n/a	n/a	n/a	n/a

^a^ Adjusted for gender and age

^b^ Respondents were able to have separate complaints at the same time

RR = relative risk; CI = confidence interval; n/a = not applicable

The univariable analyses did not show an elevated risk for exposed individuals who ingested water during swimming (RR = n/a; p-value = n/a for less than three sips of water, and p-value = 0.18 for three sips or more). The multivariable analysis, which included the variable ingestion of water, age, and gender, indicated that participants of the event of 35 years and older had an increased risk for AGI compared to those under 35 years of age (RR = 8.4; 95% CI: 1.07–66.7) (Table 2).

**Table 2:** Univariable and multivariable analysis on risk factors for acute gastrointestinal illness in the Utrecht SingelSwim 2016 among the exposed group.

Characteristic	Total N	Cases (attack rate)	Univariable		Multivariable	
			RR (95% CI)	P-value	aRR (95% CI)^a^	P-value
Gender						
• Female	88	4 (4.6%)	1	-	1	-
• Male	72	4 (5.6%)	1.2 (0.32–4.7)	0.77	1.1 (0.31–4.2)	0.85
Age (years)						
• <35	93	1 (1.1%)	1	-	1	-
• = >35	67	7 (10.5%)	9.7 (1.2–77)	0.007	8.4 (1.1–67)	0.043
Water ingested						
• No	30	0 (0%)	*n/a*	*n/a*	*n/a*	
• Yes	130	8 (6.2%)				

^a^ Adjusted for any exposure with p-value <0.2 in the univariable analysis and age

N: number of subjects who were exposed to the exposure category. Cases: Number with gastrointestinal complaints in week after event who had been exposed to the exposure category.

RR = relative risk; CI = confidence interval; n/a = not applicable

### Amsterdam City Swim cohort

**Fig 4:**
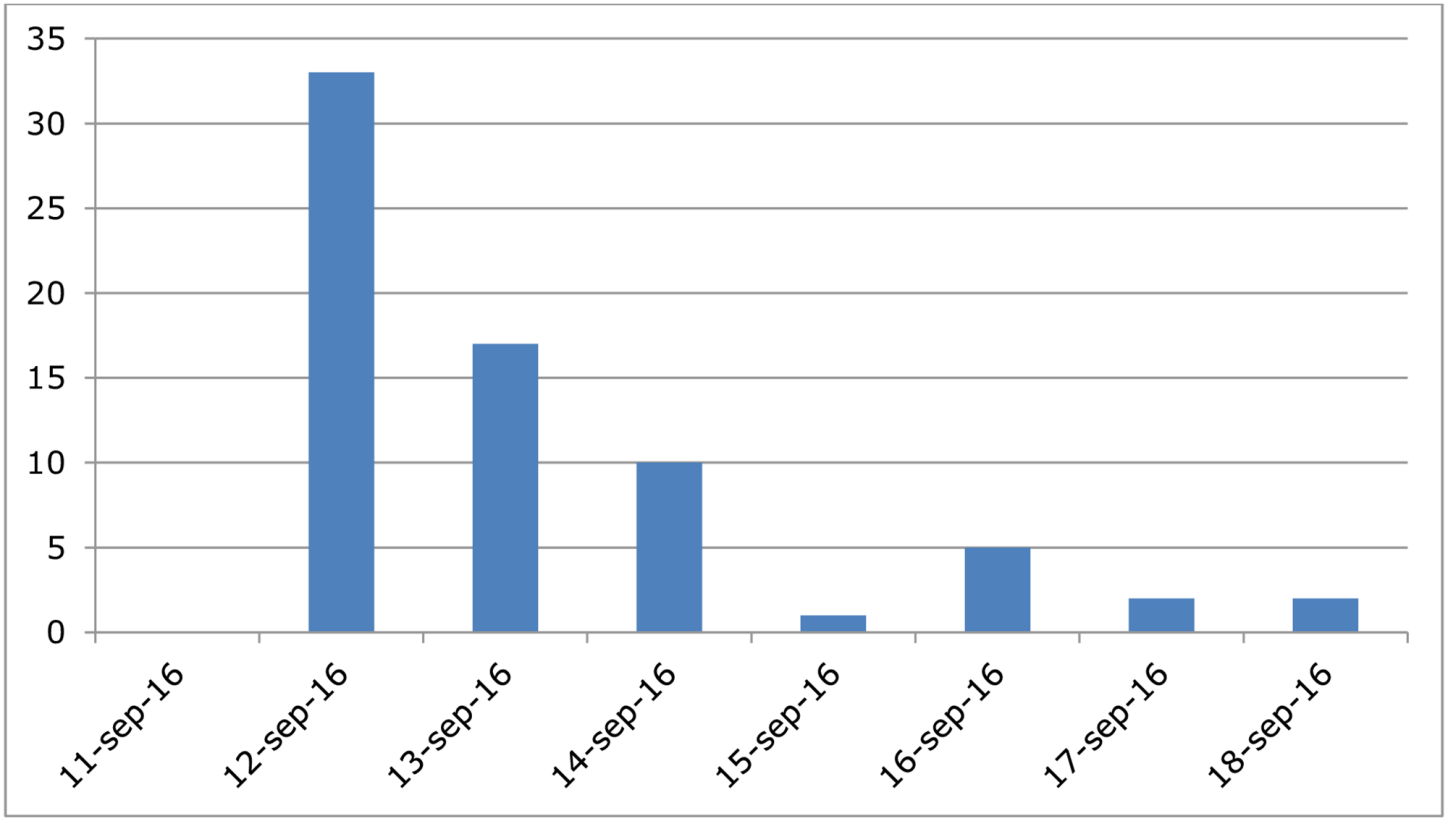
Epidemiological curve Amsterdam City Swim (ACS), 11 September 2016. The number of cases by date of onset of Acute Gastrointestinal Illness following the event.

A total of 1,169 ACS participants responded to the questionnaire (response rate 43%). From relatives and friends who did not participate in the event, we received 410 questionnaires. Comparison of demographic characteristics (age, presence of chronic disease, and gender) indicate a significant difference between exposed and non-exposed respondents for age (mean 40 years; CI: 40–42 and mean 44 years; CI: 43–46 respectively, p-value = 0.01), but not for chronic disease and gender (p-value = 0.14 and p-value = 0.92, respectively). A total of 212 (18%) of the exposed and 31 (8%) of the non-exposed reported any health complaint after the event (acute gastrointestinal illness, respiratory illness, skin complaints). Exposed individuals were more at risk to develop any health complaints after the event (p-value < 0.001). Cases of gastrointestinal, respiratory, and skin symptoms were also compared between exposed and non-exposed (Table 3). AGI (n = 71; 6.1%) and respiratory illness (n = 52; 4.5%) were the most reported health complaint among exposed and there were significantly more cases of AGI and respiratory illness among exposed compared to non-exposed. Most AGI cases developed symptoms one day after the event (Fig 4).

**Table 3:** Reported health complaints of questionnaire respondents of the Amsterdam City Swim 2016.

	Exposed		Non-exposed		Univariable RR (95% CI)	P-value	Multivariable RR (95% CI)^a^	P-value
	(N = 1169) Cases n	%	(N = 410) Cases n	%				
**Health complaints** ^b^								
Acute gastrointestinal illness	71	6.1	5	1.2	5.0 (2.0–12	<0.001	4.9 (2.0–12)	0.001
Respiratory illness	52	4.5	7	1.7	2.6 (1.2–5.7)	0.012	2.5 (1.2–5.5)	0.020
Skin symptoms	3	0.3	0	0	n/a	n/a	n/a	n/a

^a^ Adjusted for gender and age

^b^ Respondents were able to have separate complaints at the same time

RR = relative risk; CI = confidence interval; n/a = not applicable

In the univariable analysis no variable was found to be associated with an increased risk of AGI other than female gender (RR = 0.55). No elevated risk for AGI was found for the exposed individuals who ingested water (RR = 1.3). The amount of water ingested was also not associated with AGI. Swallowing less than tree sips of water compared to no water ingestion and more than tree sips of water compared to water ingestion both did not result in higher risks (RR 1.4; 95% CI: 0.72–2.6 and p-value = 0.3 for less than three sips, RR 1.3; 95% CI: 0.71–2.4 and p-value = 0.4 for three sips or more).

In the multivariable analysis males had a lower risk for AGI than females (RR 0.58; 95% CI: 0.35–0.96 and p-value = 0.017) (Table 4). Exposed individuals with cardiovascular disease had a higher risk for respiratory complaints after participating in the ACS (RR 4.9; 95% CI: 1.7–14 and p-value = 0.003).

**Table 4:** Univariable and multivariable analysis of risk factors for acute gastrointestinal illness in the Amsterdam City Swim 2016 among participants of the event.

Characteristic	Total N	Cases (attack rate)	Univariable		Multivariable	
			RR (95% CI)	P-value	RR (95% CI)^a^	P-value
Gender						
• Female	664	50 (7.5%)	1		1	
• Male	505	21 (4.2%)	0.55 (0.34–0.91)	0.017	0.58 (0.35–0.96)	0.034
Age (years)						
• <35	398	31 (7.8%)	1		1	
• = >35	771	40 (5.2%)	0.67 (0.42–1.1)	0.078	0.74 (0.47–1.2)	0.22
Water ingested						
• No	287	14 (4.9%)	1		1	
• Yes	882	57 (6.5%)	1.3 (0.75–2.3)	0.33	1.27 (0.72–2.2)	0.41
Use of any medication						
• No	968	65 (6.7%)	1		1	
• Yes	201	6 (2.9%)	0.44 (0.20–1.0)	0.044	0.45 (0.20–1.0)	0.056

^a^ Adjusted for any exposure with p-value <0.2 in the univariable analysis

N: number of subjects who were exposed to the exposure category. Cases: Number with gastrointestinal complaints in week after event who had been exposed to the exposure category. RR = relative risk; CI = confidence interval

### Laboratory results

Two out of six water samples collected during the USS tested positive for norovirus genogroup II. Reports from the urban water authorities in Utrecht showed no elevated levels of *E*.*coli* or enterococci before the USS in any of the samples taken (five days before). The cut-off values used for the qualification of good quality swimming water based on single sample results are 1800 cfu/100 mL for *E*. *coli* and 400 cfu/ 100 mL for enterococci, according to Dutch national guidelines for recreational water [20,21].

In the ACS, during the start of the event, no elevated levels of *E*.*coli* or enterococci were seen in water samples from four different locations along the route of the event. *E*.*coli* levels were between 94–981 cfu/100 mL at the different locations. Enterococci levels were 15–77 cfu/100 mL at the different locations.

The PHS contacted five participants with AGI after USS to provide stool samples. Five participants agreed on providing a stool sample, however only three participants with AGI after the USS sent in a stool sample. One sample tested positive for rotavirus. None of the participants of the event with AGI after the ACS returned a stool sample.

## Discussion

This study prospectively investigated the risk of illness among participants in two city canal swimming events. The risk of developing AGI among participants of the USS event was increased, although not statistically significant. This could be due to the small sample size. Participants of the ACS had a significantly elevated risk for developing AGI or respiratory illness after the swimming event. It is noteworthy that neither the organization nor the participants of each event reported health complaints or an outbreak to the PHS. The majority of AGI cases in both events developed health complaints within one day after the event. No risk factors for illness were identified among individuals who developed AGI after participating in the USS. For the ACS, females had a higher risk of AGI compared to the males.

Only one stool sample tested positive for a pathogen. However, several water samples tested positive. The reason to test water and stool samples was to potentially match pathogens. Due to the low number of positive stool samples, it was not possible to match pathogens between stool and water samples. Interpretation of the meaning of these positive samples is difficult. Despite the fact the samples contained norovirus, an increased risk of illness depends on virus pathogenicity, virus concentration, but also the health status of swimmers and their swimming behavior.

Laboratory investigations of the USS identified norovirus genogroup II in the collected water before and after the event. Rotavirus was identified in the stool sample of one participant of the USS with AGI, but it was unclear whether the infection occurred before, during, or after the event. The negative stool samples of exposed individuals with AGI had their stool collected a week (or more) after their symptoms had stopped, which hampered the possibility to detect microbial agents. Due to different pathogens detected in water and stool samples, no serotype matching could be performed.

For the ACS, none of the three participants who were contacted by the PHS handed in a stool sample. Therefore the water collected during the ACS was not further tested. It was difficult to obtain stool samples because not all respondents provided their contact details. Our priority was a high response rate of the questionnaire and therefore we chose to keep provision of contact details optional and to otherwise keep respondents anonymous. As a result we could only contact three persons in total from two events with AGI to collect stool samples. It is likely that most of the cases had a viral infection, since most cases developed symptoms one day after the event. Bacterial gastroenteritis typically has a longer incubation period than viral gastrointestinal disease [23,24].

Results from the current ACS study, with an attack rate of AGI of 6%, are consistent with two earlier studies of swimming events in the Netherlands [11,12]. The average age of the exposed group was significantly different from the non-exposed group. This difference is clinically not relevant for the risk of AGI. However, we only compared the exposed and non-exposed group by demographic variables and chronic diseases. Not all variables that might influence the risk of acute infection after swimming were compared. This means that the risk of bias due to differences between the groups remains present.

During the 2015 edition of the ACS, an outbreak of 427 cases was reported with an attack rate of 31% [12]. Heavy rainfall followed by sewage overflow before the event was mentioned as the most likely reason for the high attack rate. One water sample and five stool samples were found positive for norovirus genogroup I. No match was found in further subtyping. Two other water samples were positive for norovirus genogroup II. Another event in 2015, a triathlon in urban water within the city of Utrecht, resulted in an outbreak of AGI with 73 cases and an attack rate of 31% [11]. Water samples in this study were PCR-positive for norovirus genogroup I and rotavirus and stool samples were PCR-positive for norovirus genogroup II. It is well known that recreation in open water can bring certain health risks. Outbreaks but also sporadic illness are no novelty after swimming in any type of open water [7,25–27].

In light of previous publications, our study also looked at pathogens and water quality.

Analyses from the water authorities in our study showed *E*.*coli* and enterococci levels below threshold regulation norms of 1800 cfu/100 mL for *E*. *coli* and 400 cfu/ 100 mL five days before the USS and levels below threshold during the ACS. There had been no heavy rainfall and therefore no sewage overflow in the days before the USS and ACS. Therefore we did not expect indicator bacteria levels to exceed thresholds during the events, although this could not be guaranteed. The water would have been considered ‘good quality swimming water’ according to EU guidelines [1] in the days before the event in the USS and during the ACS. However, bacterial parameters do not give an indication for viral pathogen levels in water nor are they an indication for the risk of health complaints after swimming in open water [28–31].

The dry weather conditions during the 2016 edition of both events could explain the absence of an outbreak of AGI.

Pathogens are always present in urban canal water but it is difficult to predict when this leads to an outbreak of AGI. In other countries, numerous outbreaks of gastroenteritis have been described after swimming in other types of open water, such as marine water or fresh water lakes [7,25,27,32,33]. Some of the outbreaks had point sources or person-to-person transmission after swimming. Thus the risks of swimming in non-monitored open water are known and have been described previously. Our study, however, is the second to explicitly assess the risks of swimming in an urban canal with no point source but several ways of contamination (sewer-overflow, houseboats, and animals) and indicator bacteria-levels below threshold. To make a decent risk assessment in the run up to a swimming event in canal water, it is therefore important to keep the environmental factors (pollution), weather conditions, and specific risk groups in mind, rather than solely measure indicator bacteria levels. PHS needs to stay in contact with organizers of events, water authorities, and local governments in order to assess all factors. It is difficult to conduct near real-time measurements of pathogens in water. There is no test available for rapid analysis of viral or bacterial pathogen levels combining fast filtration and water sample analysis within a few hours. Near real-time analysis could help in taking last-minute preventive measures before a swimming event to prevent disease outbreaks. If the risk of health problems is considered too high, events could be moved to a different location. However, it is debatable whether positive results should lead to cancellation of such an event, especially given the challenges in translating pathogen-positive results of water samples into health risks for participants. It is difficult to do a thorough analysis of environmental water. Fairly large amounts of water are needed for one sample. Between one to ten liters of water is enough to conduct reliable analyses on open water samples. Filtration and analyses with PCR are time consuming and can be expensive, and personnel are required to collect the samples. PHS’ or local water authorities do not have the capacity to do this regularly. Therefore, information about other variables is valuable for risk assessment.

Our study was conducted in an efficient manner and with relatively low costs. The study once again provides evidence of a possible elevated risk for developing AGI for participants of canal swimming events. The role of water sample analysis before canal swimming events in predicting health risks remains uncertain and needs further development. The relation between positive water samples and actual health risks needs further investigation. Swimming events in different types of water and under different weather circumstances should also be investigated, whether an outbreak is reported or not. More data could result in identifying other risk factors for which positive interventions can be designed.

This study does have limitations. First of all, the participants of the swimming events were asked to send a hyperlink to the questionnaire to their relatives. These relatives might have been non-exposed individuals who were not present at the event but were exposed to other risk factors for AGI. This results in different potential risk factors for acquiring AGI between exposed and non-exposed, which makes interpretation of the analyses more difficult. Nonetheless, this method was used because the aim was to include a large number of non-exposed. From our questionnaire we could tell whether this non-exposed respondent participated in any swimming or sports event in open water. This might have influenced their exposure risk. We took this into account in the statistical analysis to the extent possible given the limited sample size. Requesting participants of the swimming events to forward the questionnaire was not the optimal option, but this was the most suitable and time-efficient for our situation to minimize recall bias.

Exposed individuals suffering from gastrointestinal, respiratory, or skin complaints might have been more motivated to fill in the questionnaire. The number of AGI cases could, therefore, have been overestimated in both events among the exposed group, as most people will know that swimming in canals might bring health risks. Among respondents who did not participate, the attack rate is also likely to have been overestimated. We do not expect this effect to be that much higher in one group or the other. For this reason, we believe the results are comparable. To enhance response rates, also among the group who do not have health complaints, an incentive could be awarded to one of the respondents, or participants of the city swims could be asked to forward the questionnaire to more relatives and friends.

Due to the low number of respondents in the USS it is difficult to draw conclusions from statistical analysis, given the broad confidence intervals in our results. With the sample size we had in the USS, the minimum detectable effect size for the RR would be 1.4 and a proportion of cases in the exposed group of 73%. Whereas both cities and events are comparable in location and weather conditions, the inconclusive results from the USS are likely due to the smaller sample size. As expected, a larger study population (ACS 2015 and 2016 edition) will lead to more conclusive results.

More data and knowledge about risk factors or circumstances which lead to higher risk of disease would help the PHS to advise local governments, organizers of swimming events, and its participants to take specific measures. The ACS organization, for example, set up criteria for when the event in the canals of Amsterdam should take place in future editions. One of the criteria is that the event cannot take place if water of uncertain quality is used to flush and clean the canals after sewages overflow. The PHS also advised the organizers of both events to inform their participants about the health risks of swimming in canal water and advise their participants to minimize water ingestion while swimming. This information was put on the website of both events. The effect of this information provision is not known.

In summary, the risk for AGI during the ACS event was significantly higher in the exposed group (the swimmers). This was not seen in the USS, probably due to a smaller sample size. The lack of environmental and stool laboratory analyses made the cause of the AGI cases unclear. As is known, swimming in non-monitored open water can bring health risks. The risks of AGI after urban open water swimming events vary, because many factors may influence these health risks. More knowledge about environmental and human risk factors helps reduce the risk as much as possible for future events.

## Supporting information

S1 File Questionnaire Utrecht SingelSwim English. (PDF)

S2 File Questionnaire Amsterdam City Swim English. (PDF)

S3 File Questionnaire Utrecht SingelSwim Dutch. (PDF)

S4 File Questionnaire Amsterdam City Swim Dutch. (PDF)

S5 File List of variables. Variables included in statistical analysis.
(PDF)

S6 File Dataset of Utrecht SingelSwim and Amsterdam City Swim. (XLS)

## References

[1] EU (2006) Directive 2006/7/EC of the European Parliament and of the Council of 15 February 2006 concerning the management of bathing water quality and repealing Directive 76/160/EEC Official Journal of the European Union 49: 25.

[2] Schets FM, van Wijnen J.H., Schoon H., Italiaander R., Berg H.H.J.L., de Roda Husman A.M. (2007) The microbiological quality of the water in the Amsterdam canals. Amsterdam: RIVM.

[3] Schets FM, de Roda Husman AM (2014) [Infections following recreational activities in lakes, rivers and canals: present and future risks of transmission in the Netherlands]. Ned Tijdschr Geneeskd 158: A7969 25322357

[4] Fong TT, Lipp EK (2005) Enteric viruses of humans and animals in aquatic environments: health risks, detection, and potential water quality assessment tools. Microbiol Mol Biol Rev 69: 357–371. 10.1128/MMBR.69.2.357-371.2005 15944460 PMC1197419

[5] Marion JW, Lee C, Lee CS, Wang Q, Lemeshow S, et al (2014) Integrating bacterial and viral water quality assessment to predict swimming-associated illness at a freshwater beach: a cohort study. PLoS One 9: e112029 10.1371/journal.pone.0112029 25409012 PMC4237328

[6] Fewtrell L, Kay D (2015) Recreational Water and Infection: A Review of Recent Findings. Curr Environ Health Rep 2: 85–94. 10.1007/s40572-014-0036-6 25821715 PMC4371824

[7] Sartorius B, Andersson Y, Velicko I, De Jong B, Lofdahl M, et al (2007) Outbreak of norovirus in Vastra Gotaland associated with recreational activities at two lakes during August 2004. Scand J Infect Dis 39: 323–331. 10.1080/00365540601053006 17454896

[8] Harder-Lauridsen NM, Kuhn KG, Erichsen AC, Molbak K, Ethelberg S (2013) Gastrointestinal illness among triathletes swimming in non-polluted versus polluted seawater affected by heavy rainfall, Denmark, 2010–2011. PLoS One 8: e78371 10.1371/journal.pone.0078371 24244306 PMC3820603

[9] Magill-Collins A, Gaither M, Gerba CP, Kitajima M, Iker BC, et al (2015) Norovirus Outbreaks Among Colorado River Rafters in the Grand Canyon, Summer 2012. Wilderness Environ Med 26: 312–318. 10.1016/j.wem.2015.02.007 25890859

[10] Hall V, Taye A, Walsh B, Maguire H, Dave J, et al (2017) A large outbreak of gastrointestinal illness at an open-water swimming event in the River Thames, London. Epidemiol Infect 145: 1246–1255. 10.1017/S0950268816003393 28162113 PMC9507846

[11] Parkkali S, Joosten R, Fanoy E, Pijnacker R, VANB J, et al (2017) Outbreak of diarrhoea among participants of a triathlon and a duathlon on 12 July 2015 in Utrecht, the Netherlands. Epidemiol Infect: 1–9. 10.1017/S0950268817001017 PMC9203431 28511732

[12] Joosten R, Sonder G, Parkkali S, Brandwagt D, Fanoy E, et al (2017) Risk factors for gastroenteritis associated with canal swimming in two cities in the Netherlands during the summer of 2015: A prospective study. PLoS One 12: e0174732 10.1371/journal.pone.0174732 28369101 PMC5378355

[13] Verhaelen K, Bouwknegt M, Lodder-Verschoor F, Rutjes SA, de Roda Husman AM (2012) Persistence of human norovirus GII.4 and GI.4, murine norovirus, and human adenovirus on soft berries as compared with PBS at commonly applied storage conditions. Int J Food Microbiol 160: 137–144. 10.1016/j.ijfoodmicro.2012.10.008 23177054

[14] Vennema H, de Bruin E, Koopmans M (2002) Rational optimization of generic primers used for Norwalk-like virus detection by reverse transcriptase polymerase chain reaction. J Clin Virol 25: 233–235. 12367660 10.1016/s1386-6532(02)00126-9

[15] Schuurman T, de Boer RF, van Zanten E, van Slochteren KR, Scheper HR, et al (2007) Feasibility of a molecular screening method for detection of Salmonella enterica and Campylobacter jejuni in a routine community-based clinical microbiology laboratory. J Clin Microbiol 45: 3692–3700. 10.1128/JCM.00896-07 17804656 PMC2168500

[16] International Organization for Standardization (1998) ISO 7899–1 Water quality—Detection and enumeration of intestinal enterococci—Part 1: Miniaturized method (Most Probable Number) for surface and waste water. Geneva: International Organization for Standardization.

[17] International Organization for Standardization (2000) ISO 7899-2:2000 Water quality—Detection and enumeration of intestinal enterococci—Part 2: Membrane filtration method. Geneva: International Organization for Standardization.

[18] International Organization for Standardization (1998) ISO 9308-3:1998 Water quality—Detection and enumeration of Escherichia coli and coliform bacteria—Part 3: Miniaturized method (Most Probable Number) for the detection and enumeration of E. coli in surface and waste water. Geneva: International Organization for Standardization.

[19] International Organization for Standardization (2014) ISO 9308-1:2014 Water quality—Enumeration of Escherichia coli and coliform bacteria—Part 1: Membrane filtration method for waters with low bacterial background flora. Geneva: International Organization for Standardization.

[20] Stuurgroep Water (2013) Beslisnotitie werkwijze individuele metingen en meetfrequentie microbiologische parameters zwemwaterrichtlijn.

[21] KWR Watercycle Research Institute (2006) Controle zwemwaterlocaties conform de Europese zwemwaterrichtlijn.

[22] StataCorp (2013) Stata Statistical Software: Release 13. College Station, TX: StataCorp LP.

[23] Lee RM, Lessler J, Lee RA, Rudolph KE, Reich NG, et al (2013) Incubation periods of viral gastroenteritis: a systematic review. BMC Infect Dis 13: 446 10.1186/1471-2334-13-446 24066865 PMC3849296

[24] European Centre for Disease Prevention and Control (2016) Systematic review on the incubation and infectiousness/shedding period of communicable diseases in children. Stockholm: ECDC.

[25] Zlot A, Simckes M, Vines J, Reynolds L, Sullivan A, et al (2015) Norovirus outbreak associated with a natural lake used for recreation—Oregon, 2014. MMWR Morb Mortal Wkly Rep 64: 485–490. 25974632 PMC4584822

[26] Dorevitch S, Dworkin MS, Deflorio SA, Janda WM, Wuellner J, et al (2012) Enteric pathogens in stool samples of Chicago-area water recreators with new-onset gastrointestinal symptoms. Water Res 46: 4961–4972. 10.1016/j.watres.2012.06.030 22819874

[27] Schets FM, De Roda Husman AM, Havelaar AH (2011) Disease outbreaks associated with untreated recreational water use. Epidemiol Infect 139: 1114–1125. 10.1017/S0950268810002347 21062530

[28] Colford JM Jr., Wade TJ, Schiff KC, Wright CC, Griffith JF, et al (2007) Water quality indicators and the risk of illness at beaches with nonpoint sources of fecal contamination. Epidemiology 18: 27–35. 10.1097/01.ede.0000249425.32990.b9 17149140

[29] Cabelli VJ, Dufour AP, Levin MA, McCabe LJ, Haberman PW (1979) Relationship of microbial indicators to health effects at marine bathing beaches. Am J Public Health 69: 690–696. 453396 10.2105/ajph.69.7.690 PMC1619103

[30] Cabelli VJ, Dufour AP, McCabe LJ, Levin MA (1982) Swimming-associated gastroenteritis and water quality. Am J Epidemiol 115: 606–616. 7072706 10.1093/oxfordjournals.aje.a113342

[31] Haile RW, Witte JS, Gold M, Cressey R, McGee C, et al (1999) The health effects of swimming in ocean water contaminated by storm drain runoff. Epidemiology 10: 355–363. 10401868

[32] Arnold BF, Schiff KC, Griffith JF, Gruber JS, Yau V, et al (2013) Swimmer illness associated with marine water exposure and water quality indicators: impact of widely used assumptions. Epidemiology 24: 845–853. 24045718 10.1097/01.ede.0000434431.06765.4a

[33] Pruss A (1998) Review of epidemiological studies on health effects from exposure to recreational water. Int J Epidemiol 27: 1–9. 9563686 10.1093/ije/27.1.1

