# Supplementary material for: Infection risks of city canal swimming events in the Netherlands in 2016
Source: PLoS One. 2018 Jul 27;13(7):e0200616. doi: 10.1371/journal.pone.0200616 (PMC6063404; doi:10.1371/journal.pone.0200616)
Supplement: S1 File — (PDF) [file pone.0200616.s001.pdf]

Public Health Service - Study SingelSwim Utrecht – questionnaire June 2016

The PHS region Utrecht investigates if participants of the SingelSwim Utrecht have run risk on health complaints by swimming in Utrecht canals.

We hope to get more insight in the health risks of swimming in open water. Therefore a questionnaire is developed. Complaints mainly develop in between two weeks after exposure. The questions therefore relate to the period of 12th June until 3rd July . Also when you did not have had complaints, it is important to fill in this questionnaire. The questionnaire can be filled in until 5th July.

Filling in the questionnaire will take 10 minutes of your time.

1. Did you participate in SingelSwim?
2. What distance did you swim? (800, 1200, 2000 meters)
3. In how many minutes did you finish the SingelSwim?
4. Did you ingest water during swimming?
5. How many sips of water did you approximately ingest? (less than 3, 3 to 6, more than 6)
6. Did you wear diving glasses?
7. Did you wear a nose clip?
8. Did you wear earplugs?
9. Did you wear a wetsuit or a shorty?
10. How many times have you trained in open water in the 3 months before the event?
11. Did you participate in a swimming or water event in open water in the week before the USS? If yes, please fill in date and location.
12. Did you travel abroad in the week before the USS?
13. Have you had one of the complaints mentioned here in the period before the event, from Sunday 11th June until Sunday 19th June? (more options can be filled in)
    - Nausea
    - Vomiting
    - Diarrhea
    - Fever (>38,5 degrees)
    - Cold chills
    - Stomach pain
    - Headache
    - Muscle pain or arthralgia
    - Red eyes
    - Ear pain
    - Having a cold, coughing or dyspnea
    - Skin rash
    - Other complaints, namely:….
14. When did this complaint start?
15. When was this complaint over? (data options or still complaints)
16. Have you had one of the complaints mentioned here in the period after the event, from 19 June until 3rd July? (more options possible)
    - Nausea
    - Vomiting
    - Diarrhea
    - Fever (>38,5 degrees)
    - Cold chills
    - Stomach pain
    - Headache

- Muscle pain or arthralgia
- Red eyes
- Ear pain
- Having a cold, coughing or dyspnea
- Skin rash
- Other complaints, namely:….

17. When did this complaint start?
18. When was this complaint over? (data options or still complaints)
19. Have you been to a general practitioner (GP) or the hospital because of your complaints? If yes, where did you go?
20. Did the GP or the hospital send in materials for laboratory investigation? (for example stool, blood, urine, nose- or throat glue, wound liquid)
21. What kind of material was send for investigation?
22. What were the results of this test?
23. Do you give permission to contact the GP or your doctor in case we want to ask additional questions? (fill in GP/hospital details)
24. What do you think could have been the cause of your complaint?
25. Did other persons in your surrounding report any comparable complaints in the week before you started having complaints? (No, family, friends/neighbors, others….)
26. Did other persons in your surrounding report any comparable complaints in the week after you started having complaints? (No, family, friends/neighbors, others….)
27. Have you been on the event venue during the USS? (participant or supporter)
28. Did you see someone vomit on the venue?
29. Did you eat snacks that were sold on the venue? (if yes, what did you eat?)
30. Did you eat something you brought yourself on the venue? (if yes, what did you eat?)
31. Did you use the mobile showers on the venue?
32. Did you use the toilets on the venue? (if yes, when was this? More options possible)
33. Do you use antacids? Namely:
34. Do you use medication of which you know reduces your immunity? Namely:
35. Do you use other medication? Namely:
36. In the previous year, have you had any of these chronic diseases? (more options can be filled in)
    - No, none
    - Absence of the spleen
    - Diabetes mellitus
    - Rheuma/ rheumatic arthritis
    - Liver disease, namely:
    - Kidney disease, namely:
    - Cardiovascular disease
    - Leukemia or other cancer type, namely:
    - Immunodeficiency, namely:
    - Lung disease, namely:
    - Disease of the gastrointestinal tract, namely:
    - Hay fever or other allergy, namely:
    - Skin disease or open wounds, namely:
    - Transplantation, namely:
    - Received in last 3 months immunoglobulins or blood transfusion, namely:
    - Another (severe) disease, namely:

37. What is your age? (in whole years)
38. What is your gender?
39. What is your length? (in whole centimeters)
40. What is your weight? (in whole kilograms)

41. How much time do you spend on physical exercise (sports) in a week? (in whole hours)
42. Do you eat meat, fish, diary products and egg? Several options possible
43. Do you use nutritional supplements? (vitamins, proteins, minerals, probiotics)
44. Do you give permission to the PHS to contact you when we have additional questions?
45. If yes, what is your telephone number and email address?
46. What is your surname?
47. What are the numbers of your postal code?
48. Do you have questions or remarks?
