# Supplementary material for: Infection risks of city canal swimming events in the Netherlands in 2016
Source: PLoS One. 2018 Jul 27;13(7):e0200616. doi: 10.1371/journal.pone.0200616 (PMC6063404; doi:10.1371/journal.pone.0200616)
Supplement: S2 File — (PDF) [file pone.0200616.s002.pdf]

Public Health Service - Study Amsterdam City Swim – questionnaire September 2016

Thank you for participation!
Please fill in the questionnaire before 1st october 2016.
This questionnaire is filled in by participants and non-participants of the Amsterdam City Swim.
The questions relate to the period of Sunday 11th September until 25th september. Filling in will take 5 till 10 minutes from your time. The questionnaire includes 109 questions.

1. Did you participate in the Amsterdam City Swim?
2. What distance did you swim? (700, 1500, 2000)
3. How many minutes did you swim? (…)
4. Do you have a good condition? (for example do you sport more than a few times a week)
5. Where you trained to swim 700/1500/2000 meters in the open air?
6. How often did you, in the 3 months before the event, swim 700/1500/2000 meters in a stretch?
7. Did you wear a wetsuit during swimming?
8. What kind of wetsuit did you wear? (shorty, long john, full wetsuit, other, namely…)
9. How did your wetsuit fit to your body? (good, to wide, to tight)
10. Which technique did you use while swimming? (breaststroke, freestyle, other, namely….)
11. Did you, accidentally, ingest water while swimming?
12. How many sips do you think you have swallowed? (0, 1, 2, 3, 4-5, 6-9, 10 or more)
13. Did you swim in open water in the week previous to the event?
14. Did you have any health complaints in the period after the event until now?
15. Have you had one of the complaints mentioned here in the period from 11 September until now? (more options can be filled in)
    - Nausea
    - Vomiting
    - Headache
    - Fever (>38 degrees)
    - Cold chills
    - Stomach pain
    - Diarrhea
    - Muscle pain or arthralgia
    - Red eyes
    - Ear pain
    - Having a cold, coughing or dyspnea
    - Skin rash
    - Symptoms of hypothermia (i.e. shivering, lethargy, drowsiness, pale skin, sleepiness, slow pulse and breathing)
    - Other complaints, namely:….
16. When did this complaint start?
17. Do you still have this complaint?
18. When was this complaint over?
19. In case of diarrhea:
    -how often in 24 hours did you had complaints of diarrhea?
20. In case of hypothermia:
    -did you had this before?, if so when?
    -Were you taken out of the water due to hypothermia?
21. Have you been to a general practitioner (GP) because of your complaints?

22. Did the GP send in materials for laboratory investigation? (for example stool, blood, urine, nose- or throat glue, wound liquid)
23. What kind of material was send for investigation?
24. What were the results of this test?
25. Do you give permission to contact the GP in case we want to ask additional questions? (fill in GP details)
26. What do you think could have been the cause of your complaint?
27. Did other persons in your surrounding report any comparable complaints in the week before you started having complaints? (No, family, friends/neighbors, others….)
28. Did other persons in your surrounding report any comparable complaints in the week after you started having complaints? (No, family, friends/neighbors, others….)
29. Have you been abroad in the week previous to the event?
30. Which country have you been visiting?
31. Do you have any of these chronic diseases? (more options can be filled in)
    - No, none
    - Absence of the spleen
    - Diabetes Mellitus
    - Rheuma/ rheumatic arthritis
    - Liver disease, namely:
    - Kidney disease, namely:
    - Cardiovascular disease
    - Leukemia or other cancer type, namely:
    - Immunodeficiency, namely:
    - Lung disease, namely:
    - Disease of the gastrointestinal tract, namely:
    - Hay fever or other allergy, namely:
    - Skin disease or open wounds, namely:
    - Transplantation, namely:
    - Received in last 3 months immunoglobulins, namely:
    - Received in last 3 months blood transfusion, namely:
    - Another (severe) disease, namely:
32. Do you use any medication?
33. Do you use medication of which you know it reduces your immunity? Namely:
34. Do you use antacids? Namely:
35. What is your age?
36. What is your gender?
37. What is your length (in cm)?
38. What is your weight (in kg)?
39. What are the first numbers of your postal code?
40. Did you use the catering service on the event?
41. Do you give permission to the PHS to contact you when we have additional questions?
42. If yes, what is your name and surname?
43. What is telephone number and email address?
