# Supplementary material for: Infection risks of city canal swimming events in the Netherlands in 2016
Source: PLoS One. 2018 Jul 27;13(7):e0200616. doi: 10.1371/journal.pone.0200616 (PMC6063404; doi:10.1371/journal.pone.0200616)
Supplement: S3 File — (PDF) [file pone.0200616.s003.pdf]

Introductie

- Fijn dat u mee wilt doen!
- De vragenlijst moet **ingevuld** worden **vóór 5 juli**
- Ben u jonger dan 16 jaar? Vul dan de lijst in samen met een ouder/verzorger/begeleider
- De vragen hebben betrekking op de periode van zondag 12 juni tot en met 3 juli
- Het invullen van de vragenlijst zal ongeveer 10 minuten duren

1. Heeft u deelgenomen aan de Singel Swim op 19 juni 2016?

☐ Ja
☐ Nee, ik ben gevraagd deze lijst als controlepersoon in te voeren ➡ Ga verder met vraag 11.

2. Welke afstand heeft u gezwommen?

☐ 1,2 kilometer
☐ 2 kilometer
☐ KidsSwim 800 meter

3. Hoeveel minuten (afgerond naar boven) heeft u over de Singel Swim gedaan?

☐☐☐

4. Heeft u mogelijk water ingeslikt?

☐ Ja
☐ Nee ➡ Ga verder met vraag 6.

5. Hoeveel slokken water schat u te hebben ingeslikt?

☐ Minder dan 3 slokken
☐ Tussen de 3 en 6 slokken
☐ Meer dan 6 slokken

6. Heeft u een duikbril gedragen?

☐ Ja
☐ Nee

7. Heeft u een neusklem gedragen?

☐ Ja
☐ Nee

8. Heeft u oordopjes gedragen?

☐ Ja
☐ Nee

9. Heeft u een wetsuit of shorty gedragen?

☐ Wetsuit
☐ Shorty

10. Hoe vaak heeft u in de 3 maanden voorafgaande aan de Singel Swim in open water getraind?

☐☐

11. Heeft u in de week van **11 t/m 18 juni (de week voor de Singel Swim)** deelgenomen aan een evenement, dat vergelijkbaar is met Singel Swim?

(bijvoorbeeld: een mudrun, of in open water gezwommen)

☐ Ja, namelijk (datum en locatie invullen):

☐ Nee

12. Bent u in de week van 11 t/m 18 juni in het buitenland geweest?

☐ Ja, namelijk in... (naam land)

➡ Ga verder met vraag *Klachten voorafgaand aan de Singel Swim*

☐ Nee ➡ Ga verder met vraag *Klachten voorafgaand aan de Singel Swim*

Het volgende deel van de vragenlijst gaat over eventuele klachten in de week **voorafgaand** aan de Singel Swim**, in de periode van zaterdag 11 tot en met zondag 19 juni**

13. Heeft u in week van **zaterdag 11 t/m zondag 19 juni (de week voor de Singel Swim)** last gehad van gezondheidsklachten?

☐ ja
☐ nee ➡ Ga verder met vraag *Klachten na afloop van de Singel Swim*

14. Welke klachten waren dat? (meerdere antwoorden mogelijk)

☐ misselijkheid
☐ overgeven
☐ diarree
☐ koorts (meer dan 38,5°C)
☐ koude rillingen
☐ buikpijn
☐ hoofdpijn
☐ spier− of gewrichtspijn
☐ rode/gevoelige ogen
☐ oorpijn
☐ verkoudheid, hoesten of benauwdheid
☐ rode bultjes op de huid of andere huidafwijkingen
☐ anders, namelijk

15. Wanneer begonnen deze klachten?

- ☐ zaterdag 11 juni
- ☐ zondag 12 juni
- ☐ maandag 13 juni
- ☐ dinsdag 14 juni
- ☐ woensdag 15 juni
- ☐ donderdag 16 juni
- ☐ vrijdag 17 juni
- ☐ zaterdag 18 juni
- ☐ zondag 19 juni (dag van de Singel Swim)

16. Wanneer waren deze klachten voorbij?

- ☐ Zaterdag 11 juni ➡ Ga verder met vraag *Klachten na afloop van de Singel Swim*
- ☐ Zondag 12 juni ➡ Ga verder met vraag *Klachten na afloop van de Singel Swim*
- ☐ Maandag 13 juni ➡ Ga verder met vraag *Klachten na afloop van de Singel Swim*
- ☐ Dinsdag 14 juni ➡ Ga verder met vraag *Klachten na afloop van de Singel Swim*
- ☐ Woensdag 15 juni ➡ Ga verder met vraag *Klachten na afloop van de Singel Swim*
- ☐ Donderdag 16 juni ➡ Ga verder met vraag *Klachten na afloop van de Singel Swim*
- ☐ Vrijdag 17 juni ➡ Ga verder met vraag *Klachten na afloop van de Singel Swim*
- ☐ Zaterdag 18 juni ➡ Ga verder met vraag *Klachten na afloop van de Singel Swim*
- ☐ Ik had nog klachten tijdens de Singel Swim ➡ Ga verder met vraag *Klachten na afloop van de Singel Swim*

De volgende vragen gaan over klachten in de periode van **zondag 19 juni tot en met zondag 3 juli**, **na afloop** van de Singel Swim

17. Heeft u in de periode van **19 t/m 3 juli (de twee weken na afloop van de Singel Swim)**, last gehad van gezondheidsklachten?

- ☐ Ja
- ☐ Nee ➡ Ga verder met vraag *Blootstelling*

18. Heeft u na afloop van de Singel Swim, last gehad van misselijkheid?

- ☐ Ja
- ☐ Nee ➡ Ga verder met vraag 21.

19. Wanneer begonnen deze klachten?

- ☐ zondag 19 juni (Singel Swim)
- ☐ maandag 20 juni
- ☐ dinsdag 21 juni
- ☐ woensdag 22 juni
- ☐ donderdag 23 juni
- ☐ vrijdag 24 juni
- ☐ zaterdag 25 juni
- ☐ zondag 26 juni
- ☐ maandag 27 juni
- ☐ dinsdag 28 juni
- ☐ woensdag 29 juni
- ☐ donderdag 30 juni
- ☐ vrijdag 1 juli
- ☐ zaterdag 2 juli
- ☐ zondag 3 juli

20. Wanneer waren deze klachten voorbij?

☐ Ik heb nog steeds klachten ➡ Ga verder met vraag 21.
☐ maandag 20 juni ➡ Ga verder met vraag 21.
☐ dinsdag 21 juni ➡ Ga verder met vraag 21.
☐ woensdag 22 juni ➡ Ga verder met vraag 21.
☐ donderdag 23 juni ➡ Ga verder met vraag 21.
☐ vrijdag 24 juni ➡ Ga verder met vraag 21.
☐ zaterdag 25 juni ➡ Ga verder met vraag 21.
☐ zondag 26 juni ➡ Ga verder met vraag 21.
☐ maandag 27 juni ➡ Ga verder met vraag 21.
☐ dinsdag 28 juni ➡ Ga verder met vraag 21.
☐ woensdag 29 juni ➡ Ga verder met vraag 21.
☐ donderdag 30 juni ➡ Ga verder met vraag 21.
☐ vrijdag 1 juli ➡ Ga verder met vraag 21.
☐ zaterdag 2 juli ➡ Ga verder met vraag 21.
☐ zondag 3 juli ➡ Ga verder met vraag 21.

21. Heeft u na afloop van de Singel Swim, last gehad van overgeven?

☐ Ja
☐ Nee ➡ Ga verder met vraag 24.

22. Wanneer begonnen deze klachten?

☐ zondag 19 juni (Singel Swim)
☐ maandag 20 juni
☐ dinsdag 21 juni
☐ woensdag 22 juni
☐ donderdag 23 juni
☐ vrijdag 24 juni
☐ zaterdag 25 juni
☐ zondag 26 juni
☐ maandag 27 juni
☐ dinsdag 28 juni
☐ woensdag 29 juni
☐ donderdag 30 juni
☐ vrijdag 1 juli
☐ zaterdag 2 juli
☐ zondag 3 juli

23. Wanneer waren deze klachten voorbij?

☐ Ik heb nog steeds klachten
☐ maandag 20 juni
☐ dinsdag 21 juni
☐ woensdag 22 juni
☐ donderdag 23 juni
☐ vrijdag 24 juni
☐ zaterdag 25 juni
☐ zondag 26 juni
☐ maandag 27 juni
☐ dinsdag 28 juni
☐ woensdag 29 juni
☐ donderdag 30 juni
☐ vrijdag 1 juli
☐ zaterdag 2 juli
☐ zondag 3 juli

24. Heeft u na afloop van de Singel Swim, last gehad van diarree?

☐ Ja
☐ Nee ➡ Ga verder met vraag 28.

25. Wanneer begonnen deze klachten?

☐ zondag 19 juni (Singel Swim)
☐ maandag 20 juni
☐ dinsdag 21 juni
☐ woensdag 22 juni
☐ donderdag 23 juni
☐ vrijdag 24 juni
☐ zaterdag 25 juni
☐ zondag 26 juni
☐ maandag 27 juni
☐ dinsdag 28 juni
☐ woensdag 29 juni
☐ donderdag 30 juni
☐ vrijdag 1 juli
☐ zaterdag 2 juli
☐ zondag 3 juli

26. Wanneer waren deze klachten voorbij?

☐ Ik heb nog steeds klachten
☐ maandag 20 juni
☐ dinsdag 21 juni
☐ woensdag 22 juni
☐ donderdag 23 juni
☐ vrijdag 24 juni
☐ zaterdag 25 juni
☐ zondag 26 juni
☐ maandag 27 juni
☐ dinsdag 28 juni
☐ woensdag 29 juni
☐ donderdag 30 juni
☐ vrijdag 1 juli
☐ zaterdag 2 juli
☐ zondag 3 juli

27. Hoe vaak per dag had u diarree? (op het moment met de meeste klachten)

☐ 0 tot 3 keer ➡ Ga verder met vraag 28.
☐ 3 tot 10 keer ➡ Ga verder met vraag 28.
☐ meer dan 10 keer ➡ Ga verder met vraag 28.

28. Heeft u na afloop van de Singel Swim, last gehad van koorts (meer dan 38,5°C)?

☐ Ja
☐ Nee ➡ Ga verder met vraag 31.

29. Wanneer begonnen deze klachten?

☐ zondag 19 juni (Singel Swim)
☐ maandag 20 juni
☐ dinsdag 21 juni
☐ woensdag 22 juni
☐ donderdag 23 juni
☐ vrijdag 24 juni
☐ zaterdag 25 juni
☐ zondag 26 juni
☐ maandag 27 juni
☐ dinsdag 28 juni
☐ woensdag 29 juni
☐ donderdag 30 juni
☐ vrijdag 1 juli
☐ zaterdag 2 juli
☐ zondag 3 juli

30. Wanneer waren deze klachten voorbij?

☐ Ik heb nog steeds klachten
☐ maandag 20 juni
☐ dinsdag 21 juni
☐ woensdag 22 juni
☐ donderdag 23 juni
☐ vrijdag 24 juni
☐ zaterdag 25 juni
☐ zondag 26 juni
☐ maandag 27 juni
☐ dinsdag 28 juni
☐ woensdag 29 juni
☐ donderdag 30 juni
☐ vrijdag 1 juli
☐ zaterdag 2 juli
☐ zondag 3 juli

31. Heeft u na afloop van de Singel Swim, last gehad van kouderillingen?

☐ Ja
☐ Nee ➡ Ga verder met vraag 34.

32. Wanneer begonnen deze klachten?

☐ zondag 19 juni (Singel Swim)
☐ maandag 20 juni
☐ dinsdag 21 juni
☐ woensdag 22 juni
☐ donderdag 23 juni
☐ vrijdag 24 juni
☐ zaterdag 25 juni
☐ zondag 26 juni
☐ maandag 27 juni
☐ dinsdag 28 juni
☐ woensdag 29 juni
☐ donderdag 30 juni
☐ vrijdag 1 juli
☐ zaterdag 2 juli
☐ zondag 3 juli

33. Wanneer waren deze klachten voorbij?

- [ ] Ik heb nog steeds klachten
- [ ] maandag 20 juni
- [ ] dinsdag 21 juni
- [ ] woensdag 22 juni
- [ ] donderdag 23 juni
- [ ] vrijdag 24 juni
- [ ] zaterdag 25 juni
- [ ] zondag 26 juni
- [ ] maandag 27 juni
- [ ] dinsdag 28 juni
- [ ] woensdag 29 juni
- [ ] donderdag 30 juni
- [ ] vrijdag 1 juli
- [ ] zaterdag 2 juli
- [ ] zondag 3 juli

34. Heeft u na afloop van de Singel Swim, last gehad van buikpijn?

- [ ] Ja
- [ ] Nee ➡ Ga verder met vraag 37.

35. Wanneer begonnen deze klachten?

- [ ] zondag 19 juni (Singel Swim)
- [ ] maandag 20 juni
- [ ] dinsdag 21 juni
- [ ] woensdag 22 juni
- [ ] donderdag 23 juni
- [ ] vrijdag 24 juni
- [ ] zaterdag 25 juni
- [ ] zondag 26 juni
- [ ] maandag 27 juni
- [ ] dinsdag 28 juni
- [ ] woensdag 29 juni
- [ ] donderdag 30 juni
- [ ] vrijdag 1 juli
- [ ] zaterdag 2 juli
- [ ] zondag 3 juli

36. Wanneer waren deze klachten voorbij?

- [ ] Ik heb nog steeds klachten
- [ ] maandag 20 juni
- [ ] dinsdag 21 juni
- [ ] woensdag 22 juni
- [ ] donderdag 23 juni
- [ ] vrijdag 24 juni
- [ ] zaterdag 25 juni
- [ ] zondag 26 juni
- [ ] maandag 27 juni
- [ ] dinsdag 28 juni
- [ ] woensdag 29 juni
- [ ] donderdag 30 juni
- [ ] vrijdag 1 juli
- [ ] zaterdag 2 juli
- [ ] zondag 3 juli

37. Heeft u na afloop van de Singel Swim, last gehad van hoofdpijn?

☐ Ja
☐ Nee ➡ Ga verder met vraag 40.

38. Wanneer begonnen deze klachten?

☐ zondag 19 juni (Singel Swim)
☐ maandag 20 juni
☐ dinsdag 21 juni
☐ woensdag 22 juni
☐ donderdag 23 juni
☐ vrijdag 24 juni
☐ zaterdag 25 juni
☐ zondag 26 juni
☐ maandag 27 juni
☐ dinsdag 28 juni
☐ woensdag 29 juni
☐ donderdag 30 juni
☐ vrijdag 1 juli
☐ zaterdag 2 juli
☐ zondag 3 juli

39. Wanneer waren deze klachten voorbij?

☐ Ik heb nog steeds klachten
☐ maandag 20 juni
☐ dinsdag 21 juni
☐ woensdag 22 juni
☐ donderdag 23 juni
☐ vrijdag 24 juni
☐ zaterdag 25 juni
☐ zondag 26 juni
☐ maandag 27 juni
☐ dinsdag 28 juni
☐ woensdag 29 juni
☐ donderdag 30 juni
☐ vrijdag 1 juli
☐ zaterdag 2 juli
☐ zondag 3 juli

40. Heeft u na afloop van de Singel Swim, last gehad van spier− of gewrichtspijn (anders of heftiger dan de gewone spierpijn na een dergelijk evenement)?

☐ Ja
☐ Nee ➡ Ga verder met vraag 43.

41. Wanneer begonnen deze klachten?

☐ zondag 19 juni (Singel Swim)
☐ maandag 20 juni
☐ dinsdag 21 juni
☐ woensdag 22 juni
☐ donderdag 23 juni
☐ vrijdag 24 juni
☐ zaterdag 25 juni
☐ zondag 26 juni
☐ maandag 27 juni
☐ dinsdag 28 juni
☐ woensdag 29 juni
☐ donderdag 30 juni

☐ vrijdag 1 juli
☐ zaterdag 2 juli
☐ zondag 3 juli

42. Wanneer waren deze klachten voorbij?

☐ Ik heb nog steeds klachten
☐ maandag 20 juni
☐ dinsdag 21 juni
☐ woensdag 22 juni
☐ donderdag 23 juni
☐ vrijdag 24 juni
☐ zaterdag 25 juni
☐ zondag 26 juni
☐ maandag 27 juni
☐ dinsdag 28 juni
☐ woensdag 29 juni
☐ donderdag 30 juni
☐ vrijdag 1 juli
☐ zaterdag 2 juli
☐ zondag 3 juli

43. Heeft u na afloop van de Singel Swim, last gehad van rode/gevoelige ogen?

☐ Ja
☐ Nee ➡ <u>Ga verder met vraag 46.</u>

44. Wanneer begonnen deze klachten?

☐ zondag 19 juni (Singel Swim)
☐ maandag 20 juni
☐ dinsdag 21 juni
☐ woensdag 22 juni
☐ donderdag 23 juni
☐ vrijdag 24 juni
☐ zaterdag 25 juni
☐ zondag 26 juni
☐ maandag 27 juni
☐ dinsdag 28 juni
☐ woensdag 29 juni
☐ donderdag 30 juni
☐ vrijdag 1 juli
☐ zaterdag 2 juli
☐ zondag 3 juli

45. Wanneer waren deze klachten voorbij?

☐ Ik heb nog steeds klachten
☐ maandag 20 juni
☐ dinsdag 21 juni
☐ woensdag 22 juni
☐ donderdag 23 juni
☐ vrijdag 24 juni
☐ zaterdag 25 juni
☐ zondag 26 juni
☐ maandag 27 juni
☐ dinsdag 28 juni
☐ woensdag 29 juni

- ☐ donderdag 30 juni
- ☐ vrijdag 1 juli
- ☐ zaterdag 2 juli
- ☐ zondag 3 juli

46. Heeft u na afloop van de Singel Swim, last gehad van oorpijn?

- ☐ Ja
- ☐ Nee ➡ Ga verder met vraag 49.

47. Wanneer begonnen deze klachten?

- ☐ zondag 19 juni (Singel Swim)
- ☐ maandag 20 juni
- ☐ dinsdag 21 juni
- ☐ woensdag 22 juni
- ☐ donderdag 23 juni
- ☐ vrijdag 24 juni
- ☐ zaterdag 25 juni
- ☐ zondag 26 juni
- ☐ maandag 27 juni
- ☐ dinsdag 28 juni
- ☐ woensdag 29 juni
- ☐ donderdag 30 juni
- ☐ vrijdag 1 juli
- ☐ zaterdag 2 juli
- ☐ zondag 3 juli

48. Wanneer waren deze klachten voorbij?

- ☐ Ik heb nog steeds klachten
- ☐ maandag 20 juni
- ☐ dinsdag 21 juni
- ☐ woensdag 22 juni
- ☐ donderdag 23 juni
- ☐ vrijdag 24 juni
- ☐ zaterdag 25 juni
- ☐ zondag 26 juni
- ☐ maandag 27 juni
- ☐ dinsdag 28 juni
- ☐ woensdag 29 juni
- ☐ donderdag 30 juni
- ☐ vrijdag 1 juli
- ☐ zaterdag 2 juli
- ☐ zondag 3 juli

49. Heeft u na afloop van de Singel Swim, last gehad van verkoudheid, hoesten of benauwdheid?

- ☐ Ja
- ☐ Nee ➡ Ga verder met vraag 52.

50. Wanneer begonnen deze klachten?

- ☐ zondag 19 juni (Singel Swim)
- ☐ maandag 20 juni
- ☐ dinsdag 21 juni
- ☐ woensdag 22 juni
- ☐ donderdag 23 juni
- ☐ vrijdag 24 juni
- ☐ zaterdag 25 juni
- ☐ zondag 26 juni
- ☐ maandag 27 juni
- ☐ dinsdag 28 juni
- ☐ woensdag 29 juni
- ☐ donderdag 30 juni
- ☐ vrijdag 1 juli
- ☐ zaterdag 2 juli
- ☐ zondag 3 juli

51. Wanneer waren deze klachten voorbij?

- ☐ Ik heb nog steeds klachten
- ☐ maandag 20 juni
- ☐ dinsdag 21 juni
- ☐ woensdag 22 juni
- ☐ donderdag 23 juni
- ☐ vrijdag 24 juni
- ☐ zaterdag 25 juni
- ☐ zondag 26 juni
- ☐ maandag 27 juni
- ☐ dinsdag 28 juni
- ☐ woensdag 29 juni
- ☐ donderdag 30 juni
- ☐ vrijdag 1 juli
- ☐ zaterdag 2 juli
- ☐ zondag 3 juli

52. Heeft u na afloop van de Singel Swim, last gehad van rode bultjes op de huid of andere huidafwijkingen?

- ☐ Ja
- ☐ Nee ➡ Ga verder met vraag 55.

53. Wanneer begonnen deze klachten?

- ☐ zondag 19 juni (Singel Swim)
- ☐ maandag 20 juni
- ☐ dinsdag 21 juni
- ☐ woensdag 22 juni
- ☐ donderdag 23 juni
- ☐ vrijdag 24 juni
- ☐ zaterdag 25 juni
- ☐ zondag 26 juni
- ☐ maandag 27 juni
- ☐ dinsdag 28 juni
- ☐ woensdag 29 juni
- ☐ donderdag 30 juni
- ☐ vrijdag 1 juli
- ☐ zaterdag 2 juli
- ☐ zondag 3 juli

54. Wanneer waren deze klachten voorbij?

☐ Ik heb nog steeds klachten
☐ maandag 20 juni
☐ dinsdag 21 juni
☐ woensdag 22 juni
☐ donderdag 23 juni
☐ vrijdag 24 juni
☐ zaterdag 25 juni
☐ zondag 26 juni
☐ maandag 27 juni
☐ dinsdag 28 juni
☐ woensdag 29 juni
☐ donderdag 30 juni
☐ vrijdag 1 juli
☐ zaterdag 2 juli
☐ zondag 3 juli

55. Heeft u na afloop van de Singel Swim, last gehad van andere gezondheidsklachten?

☐ Ja, namelijk ... [ ]
☐ Nee ➡ Ga verder met vraag 58.

56. Wanneer begonnen deze klachten?

☐ zondag 19 juni (Singel Swim)
☐ maandag 20 juni
☐ dinsdag 21 juni
☐ woensdag 22 juni
☐ donderdag 23 juni
☐ vrijdag 24 juni
☐ zaterdag 25 juni
☐ zondag 26 juni
☐ maandag 27 juni
☐ dinsdag 28 juni
☐ woensdag 29 juni
☐ donderdag 30 juni
☐ vrijdag 1 juli
☐ zaterdag 2 juli
☐ zondag 3 juli

57. Wanneer waren deze klachten voorbij?

☐ Ik heb nog steeds klachten
☐ maandag 20 juni
☐ dinsdag 21 juni
☐ woensdag 22 juni
☐ donderdag 23 juni
☐ vrijdag 24 juni
☐ zaterdag 25 juni
☐ zondag 26 juni
☐ maandag 27 juni
☐ dinsdag 28 juni
☐ woensdag 29 juni
☐ donderdag 30 juni
☐ vrijdag 1 juli
☐ zaterdag 2 juli

☐ zondag 3 juli

58. Bent u voor de klachten bij uw huisarts of in het ziekenhuis geweest?

☐ nee ➡ Ga verder met vraag 64.
☐ ja

59. Bent u bij de huisarts, in het ziekenhuis of bij beiden geweest?

☐ Huisarts
☐ Ziekenhuis
☐ Huisarts en ziekenhuis

60. Is er materiaal ingestuurd voor nader onderzoek (bijvoorbeeld ontlasting, bloed, urine, neus− of keelslijm, wondvocht)?

☐ Ja
☐ Nee ➡ Ga verder met vraag 64.

61. Welk soort materiaal is voor nader onderzoek opgestuurd? (meerdere antwoorden mogelijk)

☐ bloed
☐ ontlasting
☐ urine
☐ keelwat
☐ neuswat
☐ wondvocht
☐ anders, namelijk ____________

62. Wat is de uitslag van het onderzoek?

____

63. Geeft u toestemming om contact op te nemen met uw (huis)arts om na te vragen over klachten en eventuele resultaten van het onderzoek? (Zo ja, welke arts)

☐ Ja; naam en telefoonnummer van (huis)arts: ____________
☐ Nee

64. Indien u klachten heeft gehad, wat denkt u zelf dat de verklaring is/was van uw klachten? (optioneel)

____

65. Waren er in de week *voordat* u klachten kreeg, andere personen **met soortgelijke klachten i**n uw omgeving, zoals

☐ Familie (huisgenoten)
☐ Vrienden / buren
☐ Anderen, namelijk...
☐ Nee

66. Zijn er *nadat* u klachten kreeg, andere personen **met dezelfde klachten** in uw omgeving bijgekomen, zoals

☐ Familie (huisgenoten)
☐ Vrienden / buren
☐ Anderen, namelijk...
☐ Nee

In het volgende deel stellen we een aantal vragen over mogelijke blootstellingen aan infectieziekten. Niet alleen tijdens het evenement, maar ook voorafgaand en na afloop van de Singel Swim.

67. Bent u op 19 juni op het terrein van de Singel Swim geweest?
(bijvoorbeeld als deelnemer of als toeschouwer)

☐ Ja
☐ Nee ➡ Ga verder met vraag *Lichaamskenmerken*

68. Heeft u gedurende het evenement iemand zien braken?

☐ Nee
☐ Ja, namelijk (korte toelichting):

69. Heeft u gedurende de dag van de Singel Swim voedsel genuttigd bij de snack stands op het terrein?

☐ Ja,namelijk (soort voedsel):
☐ Nee, ik heb niets gegeten

70. Heeft u zelf meegebracht voedsel genuttigd gedurende het evenement?

☐ Ja, namelijk...
☐ Nee

71. Heeft u na deelname gebruik gemaakt van de mobiele douches op het evenemententerrein?

☐ Ja
☐ Nee

72. Heeft u tijdens het evenement gebruik gemaakt van de mobiele toiletten op het evenemententerrein?

☐ Ja
☐ Nee ➡ Ga verder met vraag *Lichaamskenmerken*

73. Indien u gebruik heeft gemaakt van de mobiele toiletten, op welk tijdstip was dit? (meerdere antwoorden mogelijk)

☐ Voor de start van de Singel Swim
☐ Gedurende de Singel Swim
☐ Na afloop van de Singel Swim

De volgende vragen gaan over uw gezondheid en persoonlijke kenmerken.

Van sommige medicijnen is bekend dat zij een invloed hebben op het risico op infecties. Dit zijn bijvoorbeeld bepaalde maagzuurremmers en middelen die het afweersysteem remmen.

74. Gebruikt u maagzuurremmers?

☐ Ja, namelijk ____
☐ Nee

75. Gebruikt u medicijnen waarvan u weet dat ze de afweer verminderen (zoals ontstekingremmers)?

☐ Ja, namelijk ____
☐ Nee

76. Gebruikt u andere medicijnen?

☐ nee ➡ Ga verder met vraag 77.
☐ ja, namelijk ____

77. Heeft u in het afgelopen jaar een van de volgende chronische ziekten gehad? Zo ja, welke? (*meerdere antwoorden mogelijk*)

Indien u een antwoord aankruist, vul dan ook het bijbehorende tekstvak in

☐ Nee, geen van onderstaande
☐ Diabetes/suikerziekte
☐ Afwezigheid van de milt
☐ Een leverziekte, namelijk ____
☐ Een nierziekte, namelijk ____
☐ Hart− en vaatziekten, namelijk ____
☐ Leukemie of een andere vorm van kanker, namelijk ____
☐ Immuunstoornis, namelijk ____
☐ Longziekte, namelijk ____
☐ Ziekte van het maagdarmkanaal, namelijk ____
☐ Hooikoorts of een andere allergie, namelijk ____

☐ Huidziekte of open wondjes, namelijk ______

☐ Reumatische artritis (reuma)

☐ Ik heb een transplantatie ondergaan van ______

☐ Een andere (ernstige) aandoening, namelijk ______

☐ Afgelopen 3 maanden behandeld met immunoglobuline of bloedtransfusie gehad ______

☐ Anders, namelijk ______

78. Wat is uw leeftijd (in hele jaren)?

☐☐☐

79. Wat is uw geslacht?

☐ man
☐ vrouw

80. Wat is uw lengte (in hele centimeters)?

☐☐☐

81. Wat is uw gewicht (in hele kilogrammen)?

☐☐☐

Om te weten te komen of bepaalde leefstijl gewoontes een relatie hebben met het wel of niet ontstaan van klachten, stellen we u hier nu enkele vragen over.

82. Hoeveel uur sport u gemiddeld per week (afgerond op hele uren)?

☐☐☐

83. Wat voor een dieet gebruikt u meestal in de afgelopen maand?

☐ Vlees en vis
☐ Vlees (geen vis)
☐ Vis (geen vlees)
☐ Geen vlees en geen vis (vegetarisch)
☐ Geen vlees, geen vis, geen melkproducten, wel ei
☐ Geen vlees, geen vis, geen melk− of eiproducten (veganistisch)
☐ Anders, namelijk: ______

84. Gebruikt u voedingssupplementen? (meerder antwoorden mogelijk)

☐ Nee
☐ Ja, extra vitaminen
☐ Ja, extra eiwitten
☐ Ja, extra mineralen
☐ Ja, probiotica
☐ Ja, anders. Namelijk: ______

De gegevens van de vragenlijst worden anoniem verwerkt. In sommige gevallen (bijvoorbeeld bij onduidelijkheid over gegeven antwoorden) is het handig als de GGD met u contact kan opnemen.

Indien u dat geen probleem vindt, laat dan in het volgende deel uw contactgegevens achter.

Als u liever uw contactgegevens niet achter wil laten, antwoord dan "NEE" op de eerste vraag. U gaat dan direct door naar het einde van de vragenlijst.

85. Vindt u het goed dat de GGD eventueel contact met u opneemt, als er nog vragen zijn naar aanleiding van de vragenlijst?

☐ Ja
☐ Nee ➡ Ga verder met vraag 89.

86. Wat is uw telefoonnummer en emailadres?
(Deze contactgegevens worden uitsluitend voor dit onderzoek gebruikt en zullen na afloop vernietigd worden.)

☐ Telefoonnummer waar u overdag bereikbaar bent: ____

☐ Emailadres: ____

87. Achternaam en voorletters:

____

88. Wat zijn de vier cijfers van uw postcode?

☐☐☐☐

89. Heeft u nog op− of aanmerkingen naar aanleiding van deze vragenlijst? (optioneel)

____

Hartelijk dank voor het invullen van deze vragenlijst!
