# Supplementary material for: Infection risks of city canal swimming events in the Netherlands in 2016
Source: PLoS One. 2018 Jul 27;13(7):e0200616. doi: 10.1371/journal.pone.0200616 (PMC6063404; doi:10.1371/journal.pone.0200616)
Supplement: S4 File — (PDF) [file pone.0200616.s004.pdf]

GGD Onderzoek Amsterdam City Swim 2016

GGD
Amsterdam

Fijn dat u mee wilt doen!

De vragenlijst graag invullen **vóór 1 oktober.**

Deze vragenlijst wordt ingevuld door deelnemers én niet-deelnemers aan de Amsterdam City Swim.

De vragen hebben betrekking op de periode van zondag 11 september t/m zondag 25 september.

Het invullen van de vragenlijst neemt 5 á 10 minuten van uw tijd in beslag.

Er zijn 109 vragen in deze vragenlijst

Introductie

[]

Heeft u deelgenomen aan de City Swim?

*

Kies één van de volgende mogelijkheden:

- ○ Ja
- ○ Nee, ik ben contactpersoon

Zwemmen

[]Welke afstand heeft u gezwommen? *

Beantwoord deze vraag alleen als aan de volgende condities is voldaan:
Antwoord was 'Ja' bij vraag '1 [G1Q00005]' (Heeft u deelgenomen aan de City Swim?)

Kies één van de volgende mogelijkheden:

- ○ 700 meter
- ○ 2000 meter
- ○ 1500 meter

[]

Hoeveel minuten (afgerond naar boven) heeft u over het zwemgedeelte gedaan?

*

Beantwoord deze vraag alleen als aan de volgende condities is voldaan:
Antwoord was 'Ja' bij vraag '1 [G1Q00005]' (Heeft u deelgenomen aan de City Swim?)

In dit veld mogen alleen cijfers ingevoerd worden.

Vul je antwoord hier in:

[ ]

[]

Heeft u een goede conditie? (bv. sport u meerdere keren per week?)

*

Beantwoord deze vraag alleen als aan de volgende condities is voldaan:
Antwoord was 'Ja' bij vraag '1 [G1Q00005]' (Heeft u deelgenomen aan de City Swim?)

Kies één van de volgende mogelijkheden:

- ○ Ja
- ○ Nee

[]

Was u getraind om 700 meter buiten te zwemmen?

*

Beantwoord deze vraag alleen als aan de volgende condities is voldaan:
Antwoord was '700 meter' bij vraag '2 [G1Q00006]' (Welke afstand heeft u gezwommen?)

Kies één van de volgende mogelijkheden:

- ○ Ja
- ○ Nee

[]Hoe vaak heeft u, in de 3 maanden voorafgaande aan de City Swim, 700 meter aan een stuk in open water gezwommen? *

Beantwoord deze vraag alleen als aan de volgende condities is voldaan:
Antwoord was '700 meter' bij vraag '2 [G1Q00006]' (Welke afstand heeft u gezwommen?) *en* Antwoord was 'Ja' bij vraag '5 [Getraindzwemmer]' (Was u getraind om 700 meter buiten te zwemmen?)

In dit veld mogen alleen cijfers ingevoerd worden.

Vul je antwoord hier in:

[ ]

[]

Was u getraind om 2000 meter buiten te zwemmen?

*

Beantwoord deze vraag alleen als aan de volgende condities is voldaan:
Antwoord was '2000 meter' bij vraag '2 [G1Q00006]' (Welke afstand heeft u gezwommen?)

Kies één van de volgende mogelijkheden:

- ○ Ja
- ○ Nee

[]Hoe vaak heeft u, in de 3 maanden voorafgaande aan de City Swim, 2000 meter aan een stuk in open water gezwommen? *

Beantwoord deze vraag alleen als aan de volgende condities is voldaan:
Antwoord was '2000 meter' bij vraag '2 [G1Q00006]' (Welke afstand heeft u gezwommen?) *en* Antwoord was 'Ja' bij vraag '7 [Getraindzwemmer1]' (Was u getraind om 2000 meter buiten te zwemmen?)

In dit veld mogen alleen cijfers ingevoerd worden.

Vul je antwoord hier in:

[ ]

[]

Was u getraind om 1500 meter buiten te zwemmen?

*

Beantwoord deze vraag alleen als aan de volgende condities is voldaan:
Antwoord was '1500 meter' bij vraag '2 [G1Q00006]' (Welke afstand heeft u gezwommen?)

Kies één van de volgende mogelijkheden:

- ○ Ja
- ○ Nee

[]Hoe vaak heeft u, in de 3 maanden voorafgaande aan de City Swim, 1500 meter aan een stuk in open water gezwommen? *

Beantwoord deze vraag alleen als aan de volgende condities is voldaan:
Antwoord was '1500 meter' bij vraag '2 [G1Q00006]' (Welke afstand heeft u gezwommen?) *en* Antwoord was 'Ja' bij vraag '9 [Getraindzwemmer2]' (Was u getraind om 1500 meter buiten te zwemmen?)

In dit veld mogen alleen cijfers ingevoerd worden.

Vul je antwoord hier in:

[ ]

[]Droeg u een wetsuit tijdens het zwemmen? *

Beantwoord deze vraag alleen als aan de volgende condities is voldaan:
Antwoord was 'Ja' bij vraag '1 [G1Q00005]' (Heeft u deelgenomen aan de City Swim?)

Kies één van de volgende mogelijkheden:

- ○ Ja
- ○ Nee

[]

Wat voor een soort wetsuit droeg u?

*

Beantwoord deze vraag alleen als aan de volgende condities is voldaan:
Antwoord was 'Ja' bij vraag '11 [Wetsuit]' (Droeg u een wetsuit tijdens het zwemmen?)

Kies één van de volgende mogelijkheden:

- ○ Shorty (korte mouwen en broekspijpen).
- ○ Long john (blote schouders).
- ○ Volledig wetsuit (bedekt armen en benen).
- ○ Anders, namelijk ______

[]Hoe sloot het wetsuit aan op uw lichaam? *

Beantwoord deze vraag alleen als aan de volgende condities is voldaan:
Antwoord was 'Ja' bij vraag '11 [Wetsuit]' (Droeg u een wetsuit tijdens het zwemmen?)

Kies één van de volgende mogelijkheden:

- ○ Goed
- ○ Te wijd
- ○ Te strak

[]

Welke techniek gebruikte u tijdens het zwemmen?

(Meerdere antwoorden mogelijk).

*

Beantwoord deze vraag alleen als aan de volgende condities is voldaan:
Antwoord was 'Ja' bij vraag '1 [G1Q00005]' (Heeft u deelgenomen aan de City Swim?)

Selecteer alle mogelijkheden:

- ☐ Borstcrawl
- ☐ Schoolslag
- ☐ Anders, namelijk: ______

[]Heeft u per ongeluk water ingeslikt tijdens het zwemmen? *

Beantwoord deze vraag alleen als aan de volgende condities is voldaan:
Antwoord was 'Ja' bij vraag '1 [G1Q00005]' (Heeft u deelgenomen aan de City Swim?)

Kies één van de volgende mogelijkheden:

- ○ Ja
- ○ Nee

[]Hoeveel slokken water schat u te hebben ingeslikt? *

Beantwoord deze vraag alleen als aan de volgende condities is voldaan:
Antwoord was 'Ja' bij vraag '1 [G1Q00005]' (Heeft u deelgenomen aan de City Swim?) *en* Antwoord was 'Ja' bij vraag '15 [G1Q00009]' (Heeft u per ongeluk water ingeslikt tijdens het zwemmen?)

Kies één van de volgende mogelijkheden:

- ○ 0
- ○ 1
- ○ 2
- ○ 3
- ○ 4-5
- ○ 6-9
- ○ 10 of meer

[]Heeft u in de week voorafgaand aan de City Swim in (ander) open water gezwommen? *

Kies één van de volgende mogelijkheden:

- ○ Nee
- ○ Ja, namelijk (datum en locatie invullen) [ ]

[]Heeft u in de periode van zondag 11 september tot heden last (gehad) van gezondheidsklachten? *

Kies één van de volgende mogelijkheden:

- ○ Ja
- ○ Nee

Gezondheidklachten

[]Misselijk? *

Beantwoord deze vraag alleen als aan de volgende condities is voldaan:
Antwoord was 'Ja' bij vraag '18 [G1Q000012]' (Heeft u in de periode van zondag 11 september tot heden last (gehad) van gezondheidsklachten?)

Kies één van de volgende mogelijkheden:

- Ja
- Nee

[]Wanneer begon deze klacht? *

Beantwoord deze vraag alleen als aan de volgende condities is voldaan:
Antwoord was 'Ja' bij vraag '19 [Misselijk]' (Misselijk?)

Vul een datum in:

[]Heeft u deze klacht nog steeds? *

Beantwoord deze vraag alleen als aan de volgende condities is voldaan:
Antwoord was 'Ja' bij vraag '19 [Misselijk]' (Misselijk?)

Kies één van de volgende mogelijkheden:

- Ja
- Nee

[]Wanneer was deze klacht voorbij? *

Beantwoord deze vraag alleen als aan de volgende condities is voldaan:
Antwoord was 'Ja' bij vraag '19 [Misselijk]' (Misselijk?) *en* Antwoord was 'Nee' bij vraag '21 [Misselijkheden]' (Heeft u deze klacht nog steeds?)

Vul een datum in:

[]

Overgeven?

*

Beantwoord deze vraag alleen als aan de volgende condities is voldaan:
Antwoord was 'Ja' bij vraag '18 [G1Q000012]' (Heeft u in de periode van zondag 11 september tot heden last (gehad) van gezondheidsklachten?)

Kies één van de volgende mogelijkheden:

- ○ Ja
- ○ Nee

[]Wanneer begon deze klacht? *

Beantwoord deze vraag alleen als aan de volgende condities is voldaan:
Antwoord was 'Ja' bij vraag '23 [Overgeven]' (Overgeven?)

Vul een datum in:

[]Heeft u deze klacht nog steeds? *

Beantwoord deze vraag alleen als aan de volgende condities is voldaan:
Antwoord was 'Ja' bij vraag '23 [Overgeven]' (Overgeven?)

Kies één van de volgende mogelijkheden:

- ○ Ja
- ○ Nee

[]

Hoe vaak per 24 uur heeft u klachten van overgeven?

(Indien meerdere dagen klachten, het gemiddeld aantal keren overgeven per 24 uur invoeren).

*

Beantwoord deze vraag alleen als aan de volgende condities is voldaan:
Antwoord was 'Ja' bij vraag '25 [Overgevenheden]' (Heeft u deze klacht nog steeds?)

In dit veld mogen alleen cijfers ingevoerd worden.

Vul je antwoord hier in:

[]Wanneer was deze klacht voorbij? *

Beantwoord deze vraag alleen als aan de volgende condities is voldaan:
Antwoord was 'Ja' bij vraag '23 [Overgeven]' (Overgeven?) *en* Antwoord was 'Nee' bij vraag '25 [Overgevenheden]' (Heeft u deze klacht nog steeds?)

Vul een datum in:

[ ]

[]

Hoe vaak per 24 uur had u klachten van overgeven?

(Indien meerdere dagen klachten, het gemiddeld aantal keren overgeven per 24 uur invoeren).

*****

Beantwoord deze vraag alleen als aan de volgende condities is voldaan:
Antwoord was 'Nee' bij vraag '25 [Overgevenheden]' (Heeft u deze klacht nog steeds?)

In dit veld mogen alleen cijfers ingevoerd worden.

Vul je antwoord hier in:

[ ]

[]Hoofdpijn? *

Beantwoord deze vraag alleen als aan de volgende condities is voldaan:
Antwoord was 'Ja' bij vraag '18 [G1Q000012]' (Heeft u in de periode van zondag 11 september tot heden last (gehad) van gezondheidsklachten?)

Kies één van de volgende mogelijkheden:

○ Ja

○ Nee

[]Wanneer begon deze klacht? *

Beantwoord deze vraag alleen als aan de volgende condities is voldaan:
Antwoord was 'Ja' bij vraag '29 [Hoofdpijn]' (Hoofdpijn?)

Vul een datum in:

[ ]

[]Heeft u deze klacht nog steeds? *

Beantwoord deze vraag alleen als aan de volgende condities is voldaan:
Antwoord was 'Ja' bij vraag '29 [Hoofdpijn]' (Hoofdpijn?)

Kies één van de volgende mogelijkheden:

- ○ Ja
- ○ Nee

[]Wanneer was deze klacht voorbij? *

Beantwoord deze vraag alleen als aan de volgende condities is voldaan:
Antwoord was 'Ja' bij vraag '29 [Hoofdpijn]' (Hoofdpijn?) *en* Antwoord was 'Nee' bij vraag '31 [Hoofdpijnheden]' (Heeft u deze klacht nog steeds?)

Vul een datum in:

[]Koorts >38 graden? *

Beantwoord deze vraag alleen als aan de volgende condities is voldaan:
Antwoord was 'Ja' bij vraag '18 [G1Q000012]' (Heeft u in de periode van zondag 11 september tot heden last (gehad) van gezondheidsklachten?)

Kies één van de volgende mogelijkheden:

- ○ Ja
- ○ Nee

[]Wanneer begon deze klacht? *

Beantwoord deze vraag alleen als aan de volgende condities is voldaan:
Antwoord was 'Ja' bij vraag '33 [Koorts]' (Koorts >38 graden?)

Vul een datum in:

[]Heeft u deze klacht nog steeds? *

Beantwoord deze vraag alleen als aan de volgende condities is voldaan:
Antwoord was 'Ja' bij vraag '33 [Koorts]' (Koorts >38 graden?)

Kies één van de volgende mogelijkheden:

- ○ Ja
- ○ Nee

[]Wanneer was deze klacht voorbij? *

Beantwoord deze vraag alleen als aan de volgende condities is voldaan:
Antwoord was 'Ja' bij vraag '33 [Koorts]' (Koorts >38 graden?) *en* Antwoord was 'Nee' bij vraag '35 [Koortsheden]' (Heeft u deze klacht nog steeds?)

Vul een datum in:

[]Koude rillingen? *

Beantwoord deze vraag alleen als aan de volgende condities is voldaan:
Antwoord was 'Ja' bij vraag '18 [G1Q000012]' (Heeft u in de periode van zondag 11 september tot heden last (gehad) van gezondheidsklachten?)

Kies één van de volgende mogelijkheden:

- Ja
- Nee

[]Wanneer begon deze klacht? *

Beantwoord deze vraag alleen als aan de volgende condities is voldaan:
Antwoord was 'Ja' bij vraag '37 [Kouderillingen]' (Koude rillingen?)

Vul een datum in:

[]Heeft u deze klacht nog steeds? *

Beantwoord deze vraag alleen als aan de volgende condities is voldaan:
Antwoord was 'Ja' bij vraag '37 [Kouderillingen]' (Koude rillingen?)

Kies één van de volgende mogelijkheden:

- Ja
- Nee

[]Wanneer was deze klacht voorbij? *

Beantwoord deze vraag alleen als aan de volgende condities is voldaan:
Antwoord was 'Ja' bij vraag '37 [Kouderillingen]' (Koude rillingen?) *en* Antwoord was 'Nee' bij vraag '39 [Kouderillingenheden]' (Heeft u deze klacht nog steeds?)

Vul een datum in:

[]Buikpijn? *

Beantwoord deze vraag alleen als aan de volgende condities is voldaan:
Antwoord was 'Ja' bij vraag '18 [G1Q000012]' (Heeft u in de periode van zondag 11 september tot heden last (gehad) van gezondheidsklachten?)

Kies één van de volgende mogelijkheden:

- Ja
- Nee

[]Wanneer begon deze klacht? *

Beantwoord deze vraag alleen als aan de volgende condities is voldaan:
Antwoord was 'Ja' bij vraag '41 [Buikpijn]' (Buikpijn?)

Vul een datum in:

[]Heeft u deze klacht nog steeds? *

Beantwoord deze vraag alleen als aan de volgende condities is voldaan:
Antwoord was 'Ja' bij vraag '41 [Buikpijn]' (Buikpijn?)

Kies één van de volgende mogelijkheden:

- Ja
- Nee

[]Wanneer was deze klacht voorbij? *

Beantwoord deze vraag alleen als aan de volgende condities is voldaan:
Antwoord was 'Ja' bij vraag '41 [Buikpijn]' (Buikpijn?) *en* Antwoord was 'Nee' bij vraag '43 [Buikpijnheden]' (Heeft u deze klacht nog steeds?)

Vul een datum in:

[]Diarree? *

Beantwoord deze vraag alleen als aan de volgende condities is voldaan:
Antwoord was 'Ja' bij vraag '18 [G1Q000012]' (Heeft u in de periode van zondag 11 september tot heden last (gehad) van gezondheidsklachten?)

Kies één van de volgende mogelijkheden:

- Ja
- Nee

[]Wanneer begon deze klacht? *

Beantwoord deze vraag alleen als aan de volgende condities is voldaan:
Antwoord was 'Ja' bij vraag '45 [Diarree]' (Diarree?)

Vul een datum in:

[]Heeft u deze klacht nog steeds? *

Beantwoord deze vraag alleen als aan de volgende condities is voldaan:
Antwoord was 'Ja' bij vraag '45 [Diarree]' (Diarree?)

Kies één van de volgende mogelijkheden:

- ○ Ja
- ○ Nee

[]

Hoe vaak per 24 uur heeft u klachten van diarree?

(Indien meerdere dagen klachten, het gemiddeld aantal keren diarree per 24 uur invoeren).

*

Beantwoord deze vraag alleen als aan de volgende condities is voldaan:
Antwoord was 'Ja' bij vraag '47 [Diarreeheden]' (Heeft u deze klacht nog steeds?)

In dit veld mogen alleen cijfers ingevoerd worden.

Vul je antwoord hier in:

[]Wanneer was deze klacht voorbij? *

Beantwoord deze vraag alleen als aan de volgende condities is voldaan:
Antwoord was 'Ja' bij vraag '45 [Diarree]' (Diarree?) *en* Antwoord was 'Nee' bij vraag '47 [Diarreeheden]' (Heeft u deze klacht nog steeds?)

Vul een datum in:

[]

Hoe vaak per 24 uur had u klachten van diarree?

(Indien meerdere dagen klachten, het gemiddeld aantal keren diarree per 24 uur invoeren).

*

Beantwoord deze vraag alleen als aan de volgende condities is voldaan:
Antwoord was 'Nee' bij vraag '47 [Diarreeheden]' (Heeft u deze klacht nog steeds?)

In dit veld mogen alleen cijfers ingevoerd worden.

Vul je antwoord hier in:

[ ]

[]Spier- of gewrichtspijn? *

Beantwoord deze vraag alleen als aan de volgende condities is voldaan:
Antwoord was 'Ja' bij vraag '18 [G1Q000012]' (Heeft u in de periode van zondag 11 september tot heden last (gehad) van gezondheidsklachten?)

Kies één van de volgende mogelijkheden:

- ○ Ja
- ○ Nee

[]Wanneer begon deze klacht? *

Beantwoord deze vraag alleen als aan de volgende condities is voldaan:
Antwoord was 'Ja' bij vraag '51 [Spiergewrpijn]' (Spier- of gewrichtspijn?)

Vul een datum in:

[ ]

[]Heeft u deze klacht nog steeds? *

Beantwoord deze vraag alleen als aan de volgende condities is voldaan:
Antwoord was 'Ja' bij vraag '51 [Spiergewrpijn]' (Spier- of gewrichtspijn?)

Kies één van de volgende mogelijkheden:

- ○ Ja
- ○ Nee

[]Wanneer was deze klacht voorbij? *

Beantwoord deze vraag alleen als aan de volgende condities is voldaan:
Antwoord was 'Ja' bij vraag '51 [Spiergewrpijn]' (Spier- of gewrichtspijn?) *en* Antwoord was 'Nee' bij vraag '53 [Spiergewrpijnheden]' (Heeft u deze klacht nog steeds?)

Vul een datum in:

[]Rode ogen? *

Beantwoord deze vraag alleen als aan de volgende condities is voldaan:
Antwoord was 'Ja' bij vraag '18 [G1Q000012]' (Heeft u in de periode van zondag 11 september tot heden last (gehad) van gezondheidsklachten?)

Kies één van de volgende mogelijkheden:

- Ja
- Nee

[]Wanneer begon deze klacht? *

Beantwoord deze vraag alleen als aan de volgende condities is voldaan:
Antwoord was 'Ja' bij vraag '55 [Rodeogen]' (Rode ogen?)

Vul een datum in:

[]Heeft u deze klacht nog steeds? *

Beantwoord deze vraag alleen als aan de volgende condities is voldaan:
Antwoord was 'Ja' bij vraag '55 [Rodeogen]' (Rode ogen?)

Kies één van de volgende mogelijkheden:

- Ja
- Nee

[]Wanneer was deze klacht voorbij? *

Beantwoord deze vraag alleen als aan de volgende condities is voldaan:
Antwoord was 'Ja' bij vraag '55 [Rodeogen]' (Rode ogen?) *en* Antwoord was 'Nee' bij vraag '57 [Rodeogenheden]' (Heeft u deze klacht nog steeds?)

Vul een datum in:

[]Oorpijn? *

Beantwoord deze vraag alleen als aan de volgende condities is voldaan:
Antwoord was 'Ja' bij vraag '18 [G1Q000012]' (Heeft u in de periode van zondag 11 september tot heden last (gehad) van gezondheidsklachten?)

Kies één van de volgende mogelijkheden:

- Ja
- Nee

[]Wanneer begon deze klacht? *

Beantwoord deze vraag alleen als aan de volgende condities is voldaan:
Antwoord was 'Ja' bij vraag '59 [Oorpijn]' (Oorpijn?)

Vul een datum in:

[]Heeft u deze klacht nog steeds? *

Beantwoord deze vraag alleen als aan de volgende condities is voldaan:
Antwoord was 'Ja' bij vraag '59 [Oorpijn]' (Oorpijn?)

Kies één van de volgende mogelijkheden:

- Ja
- Nee

[]Wanneer was deze klacht voorbij? *

Beantwoord deze vraag alleen als aan de volgende condities is voldaan:
Antwoord was 'Ja' bij vraag '59 [Oorpijn]' (Oorpijn?) *en* Antwoord was 'Nee' bij vraag '61 [Oorpijnheden]' (Heeft u deze klacht nog steeds?)

Vul een datum in:

[]Verkoudheid, hoesten of benauwdheid? *

Beantwoord deze vraag alleen als aan de volgende condities is voldaan:
Antwoord was 'Ja' bij vraag '18 [G1Q000012]' (Heeft u in de periode van zondag 11 september tot heden last (gehad) van gezondheidsklachten?)

Kies één van de volgende mogelijkheden:

- Ja
- Nee

[]Wanneer begon deze klacht? *

Beantwoord deze vraag alleen als aan de volgende condities is voldaan:
Antwoord was 'Ja' bij vraag '63 [Verkoudheid]' (Verkoudheid, hoesten of benauwdheid?)

Vul een datum in:

[]Heeft u deze klacht nog steeds? *

Beantwoord deze vraag alleen als aan de volgende condities is voldaan:
Antwoord was 'Ja' bij vraag '63 [Verkoudheid]' (Verkoudheid, hoesten of benauwdheid?)

Kies één van de volgende mogelijkheden:

- Ja
- Nee

[]Wanneer was deze klacht voorbij? *

Beantwoord deze vraag alleen als aan de volgende condities is voldaan:
Antwoord was 'Ja' bij vraag '63 [Verkoudheid]' (Verkoudheid, hoesten of benauwdheid?) *en* Antwoord was 'Nee' bij vraag '65 [Verkoudheidheden]' (Heeft u deze klacht nog steeds?)

Vul een datum in:

[]Rode bultjes op de huid? *

Beantwoord deze vraag alleen als aan de volgende condities is voldaan:
Antwoord was 'Ja' bij vraag '18 [G1Q000012]' (Heeft u in de periode van zondag 11 september tot heden last (gehad) van gezondheidsklachten?)

Kies één van de volgende mogelijkheden:

- Ja
- Nee

[]Wanneer begon deze klacht? *

Beantwoord deze vraag alleen als aan de volgende condities is voldaan:
Antwoord was 'Ja' bij vraag '67 [Rodebultjes]' (Rode bultjes op de huid?)

Vul een datum in:

[]Heeft u deze klacht nog steeds? *

Beantwoord deze vraag alleen als aan de volgende condities is voldaan:
Antwoord was 'Ja' bij vraag '67 [Rodebultjes]' (Rode bultjes op de huid?)

Kies één van de volgende mogelijkheden:

- ○ Ja
- ○ Nee

[]Wanneer was deze klacht voorbij? *

Beantwoord deze vraag alleen als aan de volgende condities is voldaan:
Antwoord was 'Ja' bij vraag '67 [Rodebultjes]' (Rode bultjes op de huid?) *en* Antwoord was 'Nee' bij vraag '69 [Rodebultjesheden]' (Heeft u deze klacht nog steeds?)

Vul een datum in:

[ ]

[]Onderkoelingsverschijnselen? (bv. rillen, sloomheid, sufheid, trage hartslag en ademhaling, slaperigheid, bleke huid). *

Beantwoord deze vraag alleen als aan de volgende condities is voldaan:
Antwoord was 'Ja' bij vraag '18 [G1Q000012]' (Heeft u in de periode van zondag 11 september tot heden last (gehad) van gezondheidsklachten?)

Kies één van de volgende mogelijkheden:

- ○ Ja
- ○ Nee

[]

Welke klachten waren dit?

(Meerdere antwoorden mogelijk).

*

Beantwoord deze vraag alleen als aan de volgende condities is voldaan:
Antwoord was 'Ja' bij vraag '71 [Onderkoeling]' (Onderkoelingsverschijnselen? (bv. rillen, sloomheid, sufheid, trage hartslag en ademhaling, slaperigheid, bleke huid).)

Selecteer alle mogelijkheden:

☐ Rillen

☐ Sloomheid

☐ Sufheid

☐ Trage hartslag

☐ Trage ademhaling

☐ Slaperigheid

☐ Bleke gelaatskleur

☐ Anders, namelijk:: ____________

[]Bent u in het verleden (al eens eerder) onderkoeld geraakt? *

Beantwoord deze vraag alleen als aan de volgende condities is voldaan:
Antwoord was 'Ja' bij vraag '18 [G1Q000012]' (Heeft u in de periode van zondag 11 september tot heden last (gehad) van gezondheidsklachten?)

Kies één van de volgende mogelijkheden:

○ Ja

○ Nee

[] Onder welke omstandigheden (bij welke activiteit)? *

Beantwoord deze vraag alleen als aan de volgende condities is voldaan:
Antwoord was 'Ja' bij vraag '73 [Onderkoelingbekend]' (Bent u in het verleden (al eens eerder) onderkoeld geraakt?)

Vul je antwoord hier in:

____

[]

Bent u tijdens de City Swim uit het water gehaald i.v.m. klachten van onderkoeling?

*

Beantwoord deze vraag alleen als aan de volgende condities is voldaan:
Antwoord was 'Ja' bij vraag '71 [Onderkoeling]' (Onderkoelingsverschijnselen? (bv. rillen, sloomheid, sufheid, trage hartslag en ademhaling, slaperigheid, bleke huid).)

Kies één van de volgende mogelijkheden:

- ○ Ja
- ○ Nee

[]Andere klachten die u nog niet heeft kunnen invullen? *

Beantwoord deze vraag alleen als aan de volgende condities is voldaan:
Antwoord was 'Ja' bij vraag '18 [G1Q000012]' (Heeft u in de periode van zondag 11 september tot heden last (gehad) van gezondheidsklachten?)

Kies één van de volgende mogelijkheden:

- ○ Ja
- ○ Nee

[]Andere klachten, namelijk: *

Beantwoord deze vraag alleen als aan de volgende condities is voldaan:
Antwoord was 'Ja' bij vraag '76 [Anders]' (Andere klachten die u nog niet heeft kunnen invullen?)

Vul je antwoord hier in:

[]Wanneer begon deze klacht? *

Beantwoord deze vraag alleen als aan de volgende condities is voldaan:
Antwoord was 'Ja' bij vraag '76 [Anders]' (Andere klachten die u nog niet heeft kunnen invullen?)

Vul een datum in:

[]Heeft u deze klacht nog steeds? *

Beantwoord deze vraag alleen als aan de volgende condities is voldaan:
Antwoord was 'Ja' bij vraag '76 [Anders]' (Andere klachten die u nog niet heeft kunnen invullen?)

Kies één van de volgende mogelijkheden:

- Ja
- Nee

[]Wanneer was deze klacht voorbij? *

Beantwoord deze vraag alleen als aan de volgende condities is voldaan:
Antwoord was 'Nee' bij vraag '79 [Andersheden]' (Heeft u deze klacht nog steeds?)

Vul een datum in:

Onderzoek

[]Bent u i.v.m. uw klachten bij een huisarts geweest? *

Beantwoord deze vraag alleen als aan de volgende condities is voldaan:
Antwoord was 'Ja' bij vraag '18 [G1Q000012]' (Heeft u in de periode van zondag 11 september tot heden last (gehad) van gezondheidsklachten?)

Kies één van de volgende mogelijkheden:

- ○ Ja
- ○ Nee

[]

Is er materiaal ingestuurd voor nader onderzoek (bijvoorbeeld ontlasting, bloed, urine, neus-of keelslijm, wondvocht)?

(Meerdere antwoorden mogelijk).

*

Beantwoord deze vraag alleen als aan de volgende condities is voldaan:
Antwoord was 'Ja' bij vraag '18 [G1Q000012]' (Heeft u in de periode van zondag 11 september tot heden last (gehad) van gezondheidsklachten?) *en* Antwoord was 'Ja' bij vraag '81 [G1Q000016]' (Bent u i.v.m. uw klachten bij een huisarts geweest?)

Kies één van de volgende mogelijkheden:

- ○ Ja
- ○ Nee

[]Welk soort materiaal is voor nader onderzoek opgestuurd?

Beantwoord deze vraag alleen als aan de volgende condities is voldaan:
Antwoord was 'Ja' bij vraag '18 [G1Q000012]' (Heeft u in de periode van zondag 11 september tot heden last (gehad) van gezondheidsklachten?) *en* Antwoord was 'Ja' bij vraag '82 [G1Q000017]' (Is er materiaal ingestuurd voor nader onderzoek (bijvoorbeeld ontlasting, bloed, urine, neus-of keelslijm, wondvocht)? (Meerdere antwoorden mogelijk).)

Selecteer alle mogelijkheden:

- ☐ Bloed
- ☐ Ontlasting
- ☐ Urine
- ☐ Keelwat
- ☐ Neuswat
- ☐ Wondvocht
- ☐ Anders, namelijk: ____________

[]Wat is de uitslag van het onderzoek (indien bekend)?

Beantwoord deze vraag alleen als aan de volgende condities is voldaan:
Antwoord was 'Ja' bij vraag '18 [G1Q000012]' (Heeft u in de periode van zondag 11 september tot heden last (gehad) van gezondheidsklachten?) *en* Antwoord was 'Ja' bij vraag '82 [G1Q000017]' (Is er materiaal ingestuurd voor nader onderzoek (bijvoorbeeld ontlasting, bloed, urine, neus-of keelslijm, wondvocht)? (Meerdere antwoorden mogelijk).)

Vul je antwoord hier in:

[]Geeft u toestemming om contact op te nemen met uw huisarts om na te vragen over klachten en eventuele resultaten van het onderzoek? *

Beantwoord deze vraag alleen als aan de volgende condities is voldaan:
Antwoord was 'Ja' bij vraag '18 [G1Q000012]' (Heeft u in de periode van zondag 11 september tot heden last (gehad) van gezondheidsklachten?) *en* Antwoord was 'Ja' bij vraag '81 [G1Q000016]' (Bent u i.v.m. uw klachten bij een huisarts geweest?)

Kies één van de volgende mogelijkheden:

- ○ Ja
- ○ Nee

[]Om contact op te kunnen nemen met uw huisarts, hebben wij de volgende gegevens nodig:

Beantwoord deze vraag alleen als aan de volgende condities is voldaan:
Antwoord was 'Ja' bij vraag '85 [G1Q000020]' (Geeft u toestemming om contact op te nemen met uw huisarts om na te vragen over klachten en eventuele resultaten van het onderzoek?)

	Naam huisarts(praktijk)	Telefoonnummer huisarts
Gegevens huisarts		

[]

Beantwoord deze vraag alleen als aan de volgende condities is voldaan:
Antwoord was 'Ja' bij vraag '85 [G1Q000020]' (Geeft u toestemming om contact op te nemen met uw huisarts om na te vragen over klachten en eventuele resultaten van het onderzoek?)

	Naam en voorletter(s)	Geboortedatum
Persoonsgegevens		

[]Wat denkt u zelf dat de verklaring is/was van uw klachten?

Beantwoord deze vraag alleen als aan de volgende condities is voldaan:
Antwoord was 'Ja' bij vraag '18 [G1Q000012]' (Heeft u in de periode van zondag 11 september tot heden last (gehad) van gezondheidsklachten?)

Vul je antwoord hier in:

[]

Waren er in de week *voordat* u klachten kreeg, andere personen *met soortgelijke klachten* in uw omgeving, zoals:

(Meerdere antwoorden mogelijk).

*****

Beantwoord deze vraag alleen als aan de volgende condities is voldaan:
Antwoord was 'Ja' bij vraag '18 [G1Q000012]' (Heeft u in de periode van zondag 11 september tot heden last (gehad) van gezondheidsklachten?)

Selecteer alle mogelijkheden:

- ☐ Nee
- ☐ Familie (huisgenoten)
- ☐ Vrienden/buren
- ☐ Anders, namelijk:

[]

Zijn er *nadat* u klachten kreeg, andere personen *met dezelfde klachten* in uw omgeving bijgekomen, zoals:

(Meerdere antwoorden mogelijk).

*

Beantwoord deze vraag alleen als aan de volgende condities is voldaan:
Antwoord was 'Ja' bij vraag '18 [G1Q000012]' (Heeft u in de periode van zondag 11 september tot heden last (gehad) van gezondheidsklachten?)

Selecteer alle mogelijkheden:

☐ Nee

☐ Familie (huisgenoten)

☐ Vrienden/buren

☐ Anderen, namelijk: ____________

[]Bent u in de week voorafgaand aan de City Swim in het buitenland geweest? *

Kies één van de volgende mogelijkheden:

○ Ja

○ Nee

[]Welk land heeft u bezocht?

Beantwoord deze vraag alleen als aan de volgende condities is voldaan:
Antwoord was 'Ja' bij vraag '91 [G1Q000023]' (Bent u in de week voorafgaand aan de City Swim in het buitenland geweest?)

Vul je antwoord hier in:

____

Algemene gezondheid

[]

Heeft u één van de volgende chronische ziekten? Zo ja, welke?

(Meerdere antwoorden mogelijk).

*

Vul alleen een opmerking in als je een antwoord kiest.

Selecteer alle mogelijke antwoorden en geef een toelichting:

- ☐ Nee, geen van onderstaande
- ☐ Afwezigheid van de milt
- ☐ Diabetes/suikerziekte
- ☐ Reumatische artritis (reuma)
- ☐ Een leverziekte, namelijk:
- ☐ Een nierziekte, namelijk:
- ☐ Hart- en vaatziekten, namelijk:
- ☐ Leukemie of een andere vorm van kanker, namelijk:
- ☐ Immuunstoornis, namelijk:
- ☐ Longziekte, namelijk:
- ☐ Ziekte van maagdarmkanaal, namelijk:
- ☐ Hooikoorts of een andere allergie, namelijk:
- ☐ Huidziekte of open wondjes, namelijk:
- ☐ Een transplantatie ondergaan van:
- ☐ Afgelopen 3 maanden immunoglobuline gehad:
- ☐ Afgelopen 3 maanden een bloedtransfusie gehad:

☐ Een andere (ernstige) aandoening, namelijk:

[]Gebruikt u medicijnen? *

Kies één van de volgende mogelijkheden:

- ○ Ja
- ○ Nee

[]Gebruikt u medicijnen waarvan u weet dat ze de afweer verminderen? *

Beantwoord deze vraag alleen als aan de volgende condities is voldaan:
Antwoord was 'Ja' bij vraag '94 [G1Q000026]' (Gebruikt u medicijnen?)

Kies één van de volgende mogelijkheden:

- ○ Ja
- ○ Nee
- ○ Weet niet

[]Welk(e) medicijn(en) gebruikt u?

Beantwoord deze vraag alleen als aan de volgende condities is voldaan:
Antwoord was 'Ja' bij vraag '95 [G1Q000027]' (Gebruikt u medicijnen waarvan u weet dat ze de afweer verminderen?)

Vul je antwoord hier in:

[]Gebruikt u maagzuurremmers? *

Beantwoord deze vraag alleen als aan de volgende condities is voldaan:
Antwoord was 'Ja' bij vraag '94 [G1Q000026]' (Gebruikt u medicijnen?)

Kies één van de volgende mogelijkheden:

- ○ Ja
- ○ Nee

[]Welke maagzuurremmer(s) gebruikt u?

Beantwoord deze vraag alleen als aan de volgende condities is voldaan:
Antwoord was 'Ja' bij vraag '97 [G1Q000028]' (Gebruikt u maagzuurremmers?)

Vul je antwoord hier in:

Overig

[]

Wat is uw leeftijd?

*

In dit veld mogen alleen cijfers ingevoerd worden.

Vul je antwoord hier in:

[]

Wat is uw geslacht?

*

Kies één van de volgende mogelijkheden:

- Vrouw
- Man

[]Wat is uw lengte (in cm)? *

In dit veld mogen alleen cijfers ingevoerd worden.

Vul je antwoord hier in:

[]Wat is uw gewicht (in kg)? *

In dit veld mogen alleen cijfers ingevoerd worden.

Vul je antwoord hier in:

[]

Wat zijn de eerste 4 cijfers van uw postcode?

*

In dit veld mogen alleen cijfers ingevoerd worden.

Vul je antwoord hier in:

[]

Indien u contactpersoon bent van een deelnemer aan de City Swim, vult u dan graag de achternaam en voorletter(s) van de zwemmer in:

(Deze contactgegevens worden uitsluitend voor dit onderzoek gebruikt en zullen na afloop vernietigd worden).

Vul je antwoord hier in:

[]Heeft u gebruik gemaakt van de all-inclusive catering op de dag van de City Swim? *

Kies één van de volgende mogelijkheden:

- Ja
- Nee
- N.v.t.

[]Vindt u het goed als de GGD Amsterdam eventueel contact met u opneemt, als er nog vragen zijn naar aanleiding van de vragenlijst? *

Kies één van de volgende mogelijkheden:

- Ja
- Nee

[]

Wat is uw achternaam en wat zijn uw voorletter(s)?

(Deze contactgegevens worden uitsluitend voor dit onderzoek gebruikt en zullen na afloop vernietigd worden).

Beantwoord deze vraag alleen als aan de volgende condities is voldaan:
Antwoord was 'Ja' bij vraag '106 [G1Q000029]' (Vindt u het goed als de GGD Amsterdam eventueel contact met u opneemt, als er nog vragen zijn naar aanleiding van de vragenlijst?)

Vul je antwoord hier in:

[]

Wat is uw telefoonnummer en emailadres?

(Deze contactgegevens worden uitsluitend voor dit onderzoek gebruikt en zullen na afloop vernietigd worden).

Beantwoord deze vraag alleen als aan de volgende condities is voldaan:
Antwoord was 'Ja' bij vraag '106 [G1Q000029]' (Vindt u het goed als de GGD Amsterdam eventueel contact met u opneemt, als er nog vragen zijn naar aanleiding van de vragenlijst?)

Vul alleen een opmerking in als je een antwoord kiest.

Selecteer alle mogelijke antwoorden en geef een toelichting:

- ☐ Telefoonnummer waarop u overdag bereikbaar bent
- ☐ Emailadres

[]Heeft u nog op-of aanmerkingen naar aanleiding van deze vragenlijst? (optioneel).

Vul je antwoord hier in:

GGD Amsterdam

Heeft u deelgenomen aan de City Swim stuurt u dan de email met de link naar deze vragenlijst door naar twee, liefst drie personen in uw eigen omgeving, die niet hebben deelgenomen aan de City Swim. Vraag hen eveneens de vragenlijst geheel in te vullen!

Mocht u vragen hebben dan kunt u tijdens kantooruren bellen met de GGD Amsterdam, team Infectieziekten (tel. 020-5555 337).

26-09-2015 – 09:44

Verstuur je vragenlijst
Bedankt voor je deelname aan deze vragenlijst.
