# Supplementary material for: Infection risks of city canal swimming events in the Netherlands in 2016
Source: PLoS One. 2018 Jul 27;13(7):e0200616. doi: 10.1371/journal.pone.0200616 (PMC6063404; doi:10.1371/journal.pone.0200616)
Supplement: S5 File — Variables included in statistical analysis. (PDF) [file pone.0200616.s005.pdf]

Supportive material S5

List of variables considered in the univariate analysis

- Participated in the swimming event (exposed)
- Age
- Gender
- Travel abroad
- Distance swam
- use of antacid
- use of immunosuppressive
- use of any other medication
- ingestion of water during swimming
- ingestion of water in 3 categories (none, <3,≥3)
- use of diving goggles
- use of noseclip
- use of earplugs
- long wetsuit or shorty
- trained in open water
- participated in an other open water event in the past week
- Body Mass Index in 3 categories
- Weekly hours of physical exercise in categories
- Presence of a medical condition:
    - diabetes
    - asplenia/hyposplenism
    - liver disease
    - kidney disease
    - cardiovascular disease
    - leukaemia or another kind of cancer
    - immunodeficiency
    - lung disease
    - gastrointestinal disease
    - hay fever or any kind of other allergy
    - skin disease/open wounds
    - rheumatic arthritis
    - organ transplantation in the past
    - treated with immunoglobulins or blood transfusion in the past 3 months
    - any other (chronic) disease
- seen somebody vomit at the event
- use of the toilets at the event
- use of showers at the event
- consumed a snack at the event
